# The Transacting Factor CBF-A/Hnrnpab Binds to the A2RE/RTS Element of Protamine 2 mRNA and Contributes to Its Translational Regulation during Mouse Spermatogenesis

**DOI:** 10.1371/journal.pgen.1003858

**Published:** 2013-10-17

**Authors:** Nanaho Fukuda, Tomoyuki Fukuda, John Sinnamon, Abrahan Hernandez-Hernandez, Manizheh Izadi, Chandrasekhar S. Raju, Kevin Czaplinski, Piergiorgio Percipalle

**Affiliations:** 1 Department of Cell and Molecular Biology, Karolinska Institutet, Stockholm, Sweden; 2 Program in Neuroscience, Stony Brook University Center for Nervous System Disorders, Stony Brook, New York, United States of America; 3 Department of Biochemistry and Cell Biology, Stony Brook University Center for Nervous System Disorders, Stony Brook, New York, United States of America; University of Nevada School of Medicine, United States of America

## Abstract

During spermatogenesis, mRNA localization and translation are believed to be regulated in a stage-specific manner. We report here that the Protamine2 (*Prm2*) mRNA transits through chromatoid bodies of round spermatids and localizes to cytosol of elongating spermatids for translation. The transacting factor CBF-A, also termed Hnrnpab, contributes to temporal regulation of Prm2 translation. We found that CBF-A co-localizes with the *Prm2* mRNA during spermatogenesis, directly binding to the A2RE/RTS element in the 3′ UTR. Although both p37 and p42 CBF-A isoforms interacted with RTS, they associated with translationally repressed and de-repressed *Prm2* mRNA, respectively. Only p42 was found to interact with the 5′cap complex, and to co-sediment with the *Prm2* mRNA in polysomes. In CBF-A knockout mice, expression of protamine 2 (PRM2) was reduced and the *Prm2* mRNA was prematurely translated in a subset of elongating spermatids. Moreover, a high percentage of sperm from the CBF-A knockout mouse showed abnormal DNA morphology. We suggest that CBF-A plays an important role in spermatogenesis by regulating stage-specific translation of testicular mRNAs.

## Author Summary

During eukaryotic gene expression, a fraction of newly exported mRNA molecules is transported to the cellular periphery for translation. The underlying mechanisms are not fully understood even though they likely affect specialized functions in many cell types including oligodendrocyets, neurons and germ cells. We discovered that the heterogeneous nuclear ribonucleoprotein CBF-A, interacts with a conserved sequence, the RNA trafficking sequence (RTS), located in the untranslated region of transported mRNAs. This interaction facilitates transport of myelin basic protein mRNA and dendritic mRNAs in oligodendrocytes and neurons, respectively. Here we investigated whether RTS-recognition by CBF-A coordinates transport and localized translation of the Protamine 2 mRNA in spermatogenic cells. During spermatogenesis the Protamine 2 mRNAs is synthesized and kept in a silent form to be translated at later stages. We show that by interacting with the RTS of the Protamine 2 mRNA both CBF-A isoforms contribute to regulate the transcript at the translational level. In a CBF-A knockout mouse model, we demonstrate that the interplay between the CBF-A isoforms in translation regulation of the Protamine 2 mRNA and other testicular transcripts has an impact on spermatogenesis.

## Introduction

In eukaryotic cells, nascent precursor (pre)-mRNAs are co-transcriptionally assembled into ribonucleoprotein particles (RNP). RNP assembly is mediated by heterogeneous nuclear ribonucleoproteins (hnRNPs), which associate with the transcripts, remain incorporated in mature RNPs, and in many cases, accompany newly synthesized transcripts from gene to polysomes 1–3. In the cytoplasm, certain RNPs are transported to specific cellular locations for translation and some hnRNPs play a key role, binding to specific elements within transported mRNAs [4]–[6]. In cultured oligodendrocytes, hnRNP A2 interacts with the cis-acting element of the myelin basic protein (MBP) mRNA, termed A2RE (hnRNP A2 response element) or RNA trafficking sequence (RTS), located in the 3′ untranslated region (UTR) of the transcript [7], [8]. RTS recognition by hnRNP A2 has been correlated with MBP mRNA trafficking towards myelin-forming processes and with stimulation of cap-dependent translation [9], [10]. Recently, we discovered that the RTS of the MBP mRNA is also targeted by the CArG box binding factor A (CBF-A) [11], also referred to as Hnrnpab. Recognition of the MBP mRNA RTS by CBF-A is important for MBP mRNA localization to the myelin compartment [11], which altogether suggests that RNA trafficking mechanisms are likely to be modulated by multiple transacting factors. CBF-A binding to RTS-like sequences of certain dendritic mRNAs was also found to be a requirement for activity-dependent transport to neuronal synapses [12]. How these mechanisms work and whether the two known CBF-A splice variants p42 (Hnrnpab1) and p37 (Hnrnpab2) synergize is not known [13], [14]. Nonetheless, the above observations and similar findings in *Xenopus laevis* [15]–[17] suggest that CBF-A plays a conserved function which can sort transcripts that are competent for cytoplasmic transport and local translation at specific subcellular compartments.

During the development of mammalian germ cells, the above mechanisms are important since expression of testicular transcripts is believed to be both spatially and temporally regulated (reviewed in ref. 18). The *Prm2* mRNA, encoding an essential nuclear protein expressed in mature sperm, is known to be stored as translation-incompetent mRNPs for 2 to 7 days before translation occurs [18]–[23]. Temporary storage of the translationally repressed haploid transcript may be coordinated by chromatoid bodies, perinuclear structures that are evident in round spermatids [24]–[26]. Subsequent translational de-repression often entails alterations in the length of the poly (A) tail [27]. In the case of the *Prm2* mRNA, poly (A) tail shortening represents a hallmark of the translationally active transcript [28]. What triggers poly (A) tail shortening and subsequent targeting of the transcript to the translation machinery is not fully understood but remodeling of the 3′ UTR of the transcript may play a key role in this transition since it is known to be targeted by many potential transacting factors [29], [30].

The *Prm2* mRNA has an RTS cis-acting element in the 3′ UTR, which displays high homology to the RTS in the MBP mRNA 3′UTR [7]. In the present study we therefore investigated whether CBF-A binds to the *Prm2* mRNA RTS and regulates the transcript during spermatogenesis. We discovered that both p37 and p42 CBF-A isoforms target the *Prm2* mRNA RTS in the 3′UTR. We found that p37 can interact with a translationally silenced form of the transcript. In contrast, in the translationally active *Prm2* mRNA, p37 is replaced by the p42 variant which interacts with the RTS element and directly targets the 5′ cap binding complex. Importantly, the CBF-A knockout mouse showed reduced levels and abnormal timing of *Prm2* mRNA translation. Furthermore, we found poor DNA compaction in the CBF-A-deficient sperm. We propose that the relay mechanism between p37 and p42 contributes to the *Prm2* mRNA translation regulation. This mechanism is important for spermatogenesis and may be conserved in other cell types.

## Results

### CBF-A is localized in chromatoid bodies of round spermatids in mouse testis

**Figure 1:**
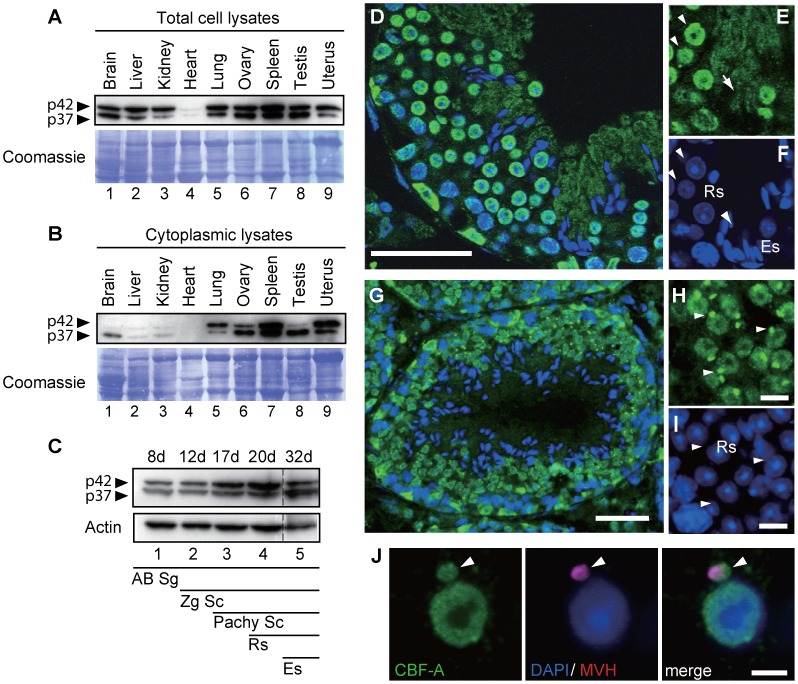
*In vivo* distribution of CBF-A in mouse testes. (**A–B**) Analysis of steady state expression levels of CBF-A on immunoblots of total (A) or cytoplasmic lysates (B) using the anti-CBF-A antibody SAK22. 20 µg proteins/lane. (**C**) Developmental expression profiles of CBF-A on immunoblots of mouse testis lysates prepared from juvenile mouse testes from days postpartum 8 (8 d) to 32 d, using the SAK22 antibody. AB Sg, AB type spermatogonia, Zg Sc, zygoten spermatocyte, Pachy Sc, Pachytene spermatocyte, Rs, Round spermatids, Es, Elongate spermatids. (**D**) Overview of mouse testes cryosections immunostained with the anti-CBF-A antibody (ICCI) and DAPI, and analyzed by confocal microscopy (see also Figure S1 for analysis with the SAK22 antibody). (**E–F**) Higher magnification images of mouse testes cryosections. (E) CBF-A is localized to nuclei of round spermatids (see arrowheads). In elongating spermatids CBF-A was preferentially found in the cytoplasm (see arrows). (F) Nuclei were stained with DAPI and shown in blue. (**G**) Overview of mouse testis cryosections subjected to antigen retrieval and immunostained using the anti-CBF-A antibody (ICCI) and DAPI. (**H–I**) Higher magnification images of a testis section stained with the ICCI antibody and DAPI. (H) The ICCI antibody labels nuclei of round spermatids as well as perinuclear compartments (see arrowheads). (**I**) Nuclei were stained with DAPI and shown in blue. (**J**) Immunostaining on squash preparations of testicular cells. The signals from the immunolabeling with the anti-CBF-A antibody (ICCI, green) and the anti-MVH antibody (magenta) were found to co-localize in chromatoid bodies of round spermatids (see arrowhead). Scale bars, D and G; 50 µm, H–J; 10 µm.

To evaluate steady state expression of CBF-A, immunoblots of total lysates from adult mouse tissues were analyzed with the anti-CBF-A antibody SAK22 that targets a conserved N-terminal epitope found in the p37 and p42 splice variants (see also Figure S1A) [12]. Both CBF-A variants were ubiquitously expressed in similar proportions with slight differences in a tissue-specific manner (Figure 1A). Analysis of the cytoplasmic fractions showed a much larger variation in the p37 to p42 ratios (Figure 1B). p37 is more abundant than p42 in the cytoplasmic fractions in brain, consistent with a previous study [31], and we observed the same pattern in both ovary and testis (Figure 1B), suggesting a possible common function of CBF-A among these three tissues.

To address the involvement of CBF-A in spermatogenesis, we next examined expressions of CBF-A in the mouse testis in more details. Immunoblots of mouse testis lysates from mice 8 dpp (days postpartum), 12 dpp, 17 dpp, 20 dpp, 32 dpp, in which Type B spermatogonia, zygotene spermatocytes, pachytene spermatocytes, round spermatids, and elongating spermatids start to appear, respectively [32], confirmed that both CBF-A isoforms are expressed from at least 8 dpp (Figure 1C). To study in vivo localization of CBF-A in spermatogenic cells, frozen testis sections were immunostained for CBF-A with the anti-CBF-A antibodies SAK22 (see Figure S1B) and ICCI (Figure 1D–J). SAK22 recognizes both splice variants, while ICCI targets a unique exon 7 found in the p42 CBF-A splice variant (Figure S1A) [11], [12]. Both antibodies confirmed that CBF-A is expressed in spermatogenic cells at all developmental stages (Figure 1D–F; Figure S1B). Throughout spermatogenesis, the CBF-A signal was detected both in nucleus and cytoplasm, however, it predominantly localized to nuclei until the round spermatid stage and to cytosol in elongating and elongated spermatids (Figure 1D–F). Furthermore, immunofluorescence staining of testis sections subjected to microwave-enhanced antigens retrieval [33] showed that CBF-A accumulates into perinuclear structures reminiscent of chromatoid bodies in round spermatids (Figure 1G–I). To further examine this peculiar distribution, we made squash preparations of seminiferous tubules, in which testicular cells are dissociated into single cells with preserved cellular structure [34]. When these preparations were analyzed by co-immunofluorescence staining with antibodies against CBF-A and the chromatoid body marker protein MVH (Mouse Vasa Homolog/DDX4) [35], [36], we found considerable overlap within chromatoid bodies (Figure 1J). Similar distributions to CBF-A were revealed for hnRNP A2 (Figure S2), demonstrating that both RTS binding proteins are in chromatoid bodies. These results raised a possibility that CBF-A is involved in the regulation of mRNA(s) that are translocated to the chromatoid bodies during spermatogenesis.

### The *Prm2* mRNA transits through chromatoid bodies during spermatogenesis

**Figure 2:**
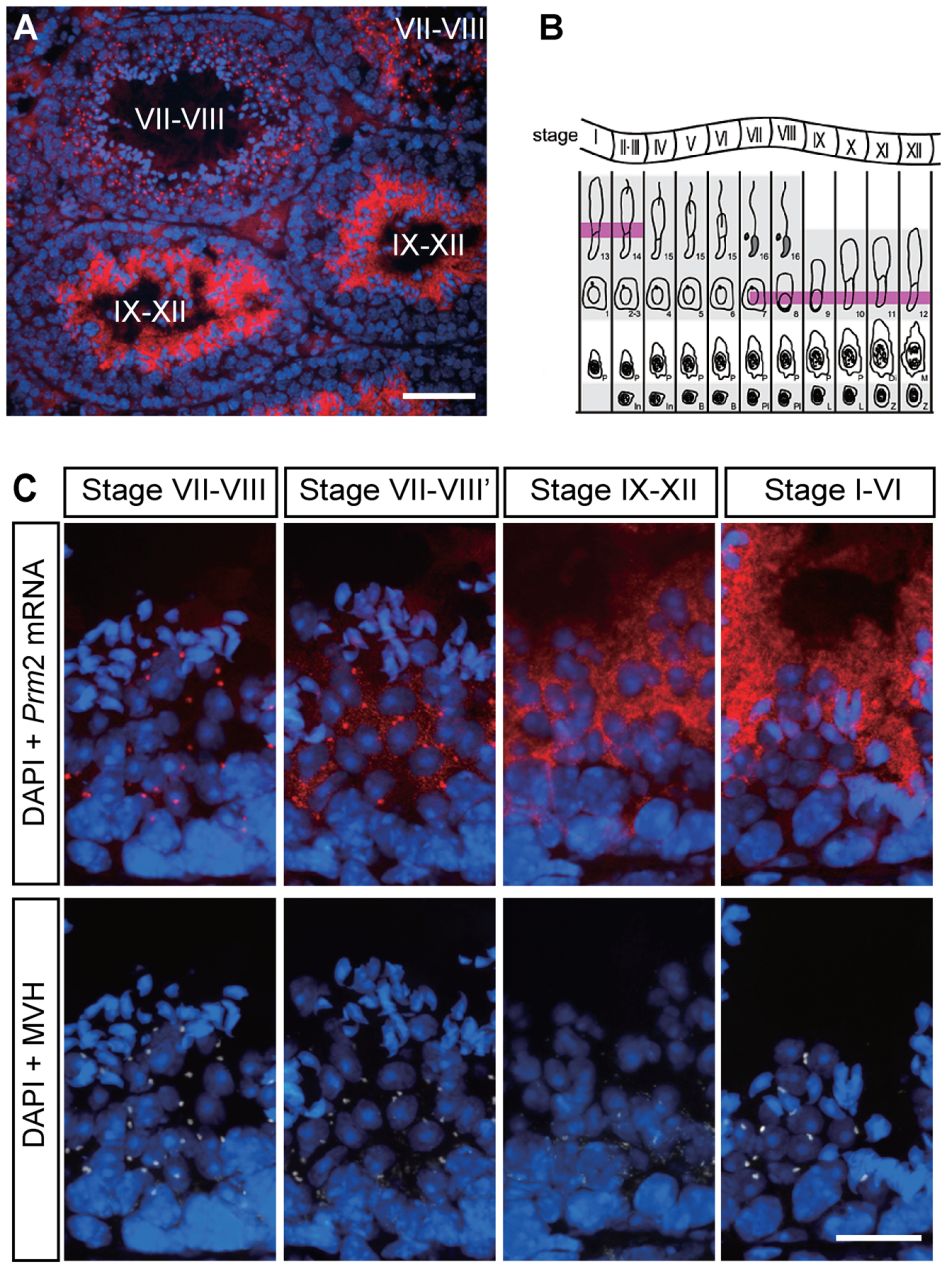
*In vivo* localization of *Prm2* mRNA in adult mouse testes. (**A**) *In situ* hybridization on testis cryosections (10 µm) using a *Prm2* mRNA antisense probe. Nuclei were stained with DAPI and shown in blue. Roman numbers indicate stages of seminiferous epithelium cycle. (**B**) Diagram of the 12-stage growth cycle of mouse spermatogenesis [55], showing the stages of the *Prm2* mRNA expression during spermatogenesis. The *Prm2* mRNA is detected from step 7 round spermatids to step 14 elongated spermatids (highlighted in magenta). (**C**) Subcellular localization of the *Prm2* mRNA in spermatids during spermatogenesis. Testis cryosections were double stained with the *Prm2* mRNA antisense probe (red, upper panels) and the anti-MVH antibody (white, lower panels). The *Prm2* mRNA was localized to perinuclear structures positive for the chromatoid body marker MVH in the spermatids at stage VII–VIII and dispersed into cytosol at later stages. Scale bar, A; 100 µm, C; 20 µm.

**Figure 3:**
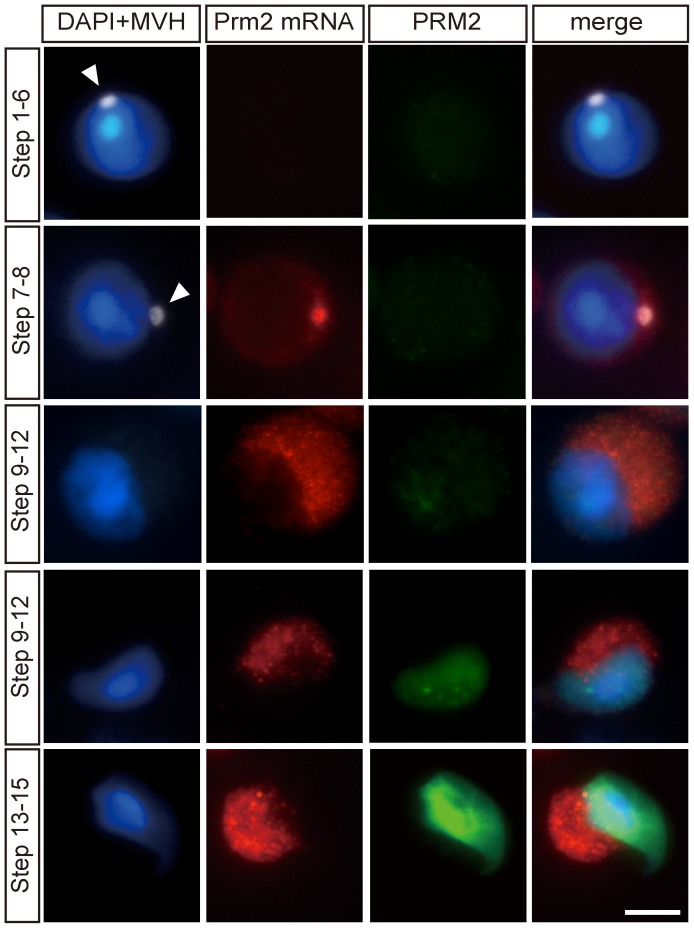
Immunostaining on tubule squash preparations of testicular cells at different steps of spermatogenesis. Cells were triple-stained with the antisense probe for the *Prm2* mRNA (red), an anti-PRM2 antibody (green) and with the anti-MVH antibody (white) as well as DAPI. The *Prm2* mRNA was co-localized with MVH to chromatoid bodies as from step 7–8 spermatids. In late step spermatids (step 9–12 and step 13–15), the *Prm2* mRNA becomes more diffusely localized to the cytosol. Scale bars, 10 µm (See also Figure S3 for more examples).

Since the 3′UTR of the *Prm2* mRNA exhibits an RTS element [7], we next asked whether the *Prm2* mRNA is a target transcript of CBF-A in spermatogenic cells. For this purpose, we first analyzed the in vivo distribution of the *Prm2* mRNA by fluorescence *in situ* hybridization on cryosections of adult mouse testis. A specific signal using a *Prm2* mRNA antisense probe was detected in post-meiotic cells, as from later step round spermatids all the way to elongating and elongated spermatids (Figure 2A–B). Control incubations with a *Prm2* mRNA sense probe gave no significant signal (Figure S3). When analyzing the *Prm2* mRNA intracellular localization, we found that in elongating and elongated spermatids, the *Prm2* mRNA is diffusely localized in the cytoplasm (Figure 2C). In contrast, in round spermatids (in stage VII–VIII), the *Prm2* transcript was highly enriched in perinuclear structures (Figure 2C). These structures were found to be positive when co-immunostained with the MVH antibody to mark chromatoid bodies (Figure 2C). On squash preparations of stage-selected segments of seminiferous tubules, we found that the *Prm2* mRNA is not expressed in step 1–6 spermatids. The *Prm2* mRNA is beginning to be expressed in step 7–8 spermatids where it is localized in chromatoid bodies and diffusely in the cytoplasm. The signal in the cytoplasm gradually increased until step 13–15 elongated spermatids where PRM2 synthesis occurs (Figures 3; Figure S4). Altogether these findings suggest that newly synthesized *Prm2* mRNA is translocated in chromatoid bodies and cytoplasm where it is stored until it is translated. The in vivo distribution of *Prm2* mRNA strikingly correlates with that of CBF-A, suggesting an involvement of CBF-A in the regulation of the *Prm2* mRNA during spermatogenesis.

### CBF-A associates with the endogenous *Prm2* mRNA

**Figure 4:**
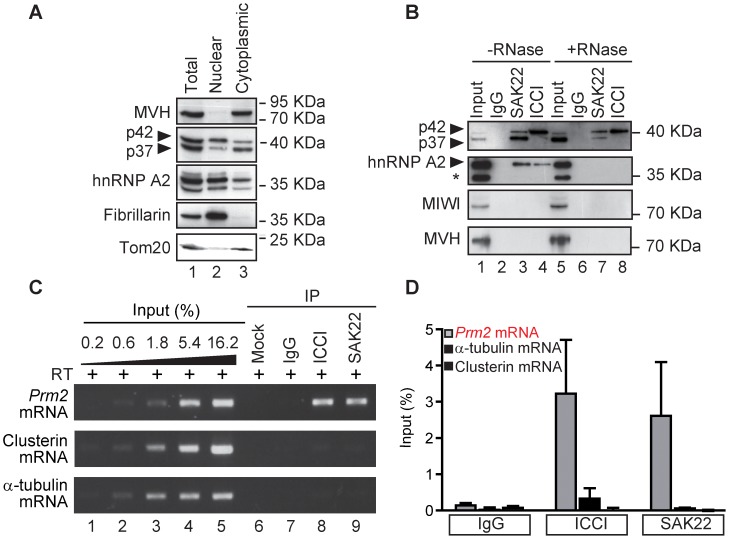
CBF-A is in cytoplasmic RNPs in complex with the *Prm2* mRNA in testicular cells. (**A**) Immunoblots of nuclear and cytoplasmic fractions of testis lysates. Anti-fibrillarin (nucleolar protein) and anti-Tom20 (mitochondrial protein) antibodies were used to characterize the fractionation. (**B**) Untreated or RNase-treated cytoplasmic fractions of testis lysates were incubated with anti-CBF-A antibodies (SAK22 or ICCI) or control non-specific IgGs and the immunoprecipitates were probed as indicated. * marks a testis-specific variant of hnRNP A2 [56]. (**C**) RNA immunoprecipitation (RIP) assays. Cytoplasmic fractions of testis lysates were incubated with anti-CBF-A antibodies (SAK22 or ICCI), control anti-mouse IgGs, or without antibodies (mock). In all cases, total RNA was extracted from the immunoprecipitates and analyzed by RT-PCR with primers amplifying *Prm2*, α-tubulin or clusterin cDNA. (**D**) Densitometric quantifications of the RIP experiments. The signal intensities of the RT-PCR bands were calculated from 3 independent experiments and shown as % of input (mean+/−SE).

We next fractionated mouse testis extracts into nuclear and cytoplasmic fractions, and analyzed the distribution of CBF-A. Immunoblotting with the SAK22 antibody revealed that CBF-A isoforms are in both subcellular fractions (Figure 4A). hnRNP A2 showed a similar distribution (Figure 4A). As expected, MVH and the mitochondrial protein Tom20 were detected in the cytoplasm, whereas fibrillarin was entirely restricted to the nuclear fraction (Figure 4A). When the cytoplasmic lysate was assayed by immunoprecipitations, SAK22 and ICCI antibodies respectively precipitated p37 as well as p42, and p42 alone (Figure 4B). Consistent with previous observations [11], hnRNPA2 was co-precipitated with CBF-A by both antibodies in an RNA-dependent manner (Figure 4B). Even though CBF-A is present in chromatoid bodies (Figure 1), neither of the two CBF-A antibodies co-precipitated MVH and the piRNA-binding protein MIWI, which localize to chromatoid bodies and are involved in germline development [37], [38] (Figure 4B). We next set out to perform RNA immunoprecipitations (RIP) to determine whether CBF-A associates with the endogenous *Prm2* mRNA. Total RNA was isolated from the protein fractions co-immunoprecipitated with the SAK22 and ICCI antibodies from testicular lysates. The precipitated RNA fractions were reverse-transcribed with oligodT primers. The resulting cDNA was analyzed by semi-quantitative PCR using primers specifically amplifying the *Prm2* cDNA. For comparison, α-tubulin and clusterin mRNAs, which are known to be translated immediately after transcription [39], were also analyzed. The results show that the *Prm2* mRNA was highly enriched in the fractions precipitated with both CBF-A antibodies (Figure 4C, see lanes 8 and 9), whereas α-tubulin and clusterin mRNAs were not significantly detected within the immunoprecipitated fractions (Figure 4C, lanes 8 and 9). In control RIPs performed in the absence of antibodies or in the presence of control IgGs, none of the transcripts analyzed was detected (Figure 4C, lanes 6 and 7). Densitometric quantifications over three independent experiments showed a specific increase in the amount of *Prm2* mRNA co-precipitated with the CBF-A antibodies (Figure 4D). We conclude that CBF-A interacts with the endogenous *Prm2* transcript in testicular cells.

### *Prm2* mRNA RTS binding by CBF-A isoforms p37 and p42 is mutually exclusive

**Figure 5:**
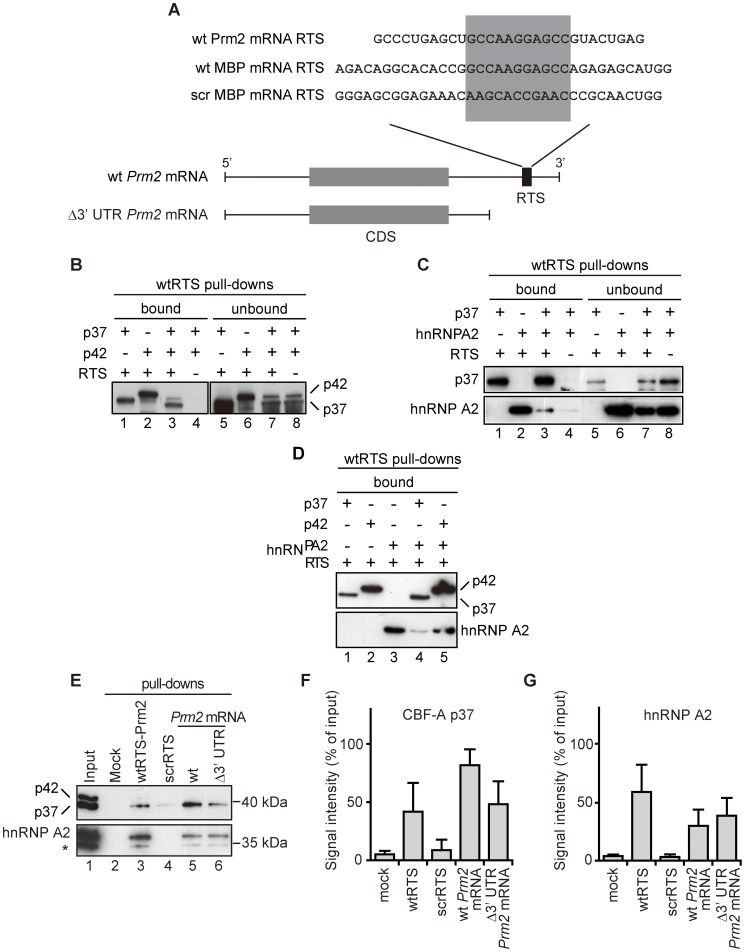
The RTS in the 3′ UTR of the *Prm2* mRNA is primarily targeted by p37. (**A**) Location and sequence of the *Prm2* mRNA RTS and control probes. For the pull-down assays all probes were biotinylated and individually coupled to streptavidin Sepharose. (**B–C**) *In vitro* RNA pull-down assays with recombinant (B) p37 and p42 or (C) p37 and hnRNP A2, incubated with wtRTS-Prm2 beads. As revealed on immunoblots with CBF-A (SAK22) or hnRNP A2 antibodies, all proteins were specifically co-precipitated with the beads. However, upon co-incubation with p37, precipitations of (B) p42 and (C) hnRNP A2 were significantly impaired. (**D**) p42 and hnRNP A2 are co-precipitated with wtRTS-Prm2 beads. (**E**) p37 specifically targets the RTS-containing *Prm2* mRNA 3′UTR. As indicated biotinylated oligonucleotides and *in vitro* transcribed full-length or Δ3′UTR *Prm2* mRNA were conjugated to Streptavidin beads and incubated with cytoplasmic testicular lysate. Bound proteins were analyzed by immunoblotting for CBF-A (SAK22 antibody) and hnRNP A2. Densitometric measurements of the bound CBF-A (**F**) and hnRNP A2 fractions (**G**) to biotinylated transcripts in panel (E) over three independent experiments are plotted as signal intensities from the respective immunoblots. The bar diagrams include standard deviations.

We next studied whether CBF-A binds to the RTS element of the *Prm2* mRNA by in vitro RNA pull down assays. For this purpose, a biotinylated RNA oligonucleotide encompassing wild-type (wt) *Prm2* mRNA RTS (wtRTS-Prm2) was conjugated to streptavidin beads (see Figure 5A). The beads were incubated with recombinant p37 and p42 as well as hnRNP A2 for comparison. After incubation with the beads, bound and unbound proteins were resolved by SDS-PAGE and visualized on immunoblots. When p37, p42 and hnRNP A2 were individually incubated with the RTS-beads, all proteins were detected in bound fractions (Figure 5B, lane 1–2; Figure 5C, lane1–2; Figure 5D, lane 1–3), indicating that p37, p42 and hnRNP A2 all have intrinsic ability to bind the RTS. However, when either p42 or hnRNP A2 were co-incubated with p37, we found that precipitations of p42 and hnRNP A2 by wtRTS-conjugated beads were significantly reduced (Figure 5B, lane 3; Figure 5C, lane3; Figure 5D, lane 4). On the contrary, when p42 and hnRNP A2 were co-incubated, both proteins were co-precipitated with wtRTS beads in similar amounts (Figure 5D, lane 5).

To find out how endogenous proteins interact with the *Prm2* mRNA, we next performed RNA-pull down assays on cytoplasmic lysates of adult mice testis. As probes, we synthesized biotinylated full length wt *Prm2* mRNA and a 3′UTR-truncated form (Δ3′ UTR *Prm2* mRNA) lacking the RTS by *in vitro* transcription (Figure 5A), and also prepared wt Prm2 mRNA RTS and a short oligonucleotide encompassing a scrambled version of the RTS (scr MBP mRNA RTS) (Figure 5A). When we analyzed bound fractions on immunoblots for p37 and p42 or hnRNP A2, we found that the p37 CBF-A variant was precipitated with wtRTS Prm2, but not significantly recovered in bound fractions with scrRTS beads or mock beads (Figure 5E, lanes 2–4). On the other hand, very low levels of p42 were co-precipitated with all RNA probes (Figure 5E). hnRNPA2 was also specifically precipitated by wtRTS Prm2. However, when analyzing the binding propensities of endogenous p37 and hnRNP A2 against the full-length *Prm2* transcript, we found that p37 was precipitated with the wt transcript more efficiently than hnRNP A2. The lack of RTS in the 3′UTR truncated Prm2 transcript led to up to 50% drop in the amount of p37 precipitated, whereas we did not observe significant reduction in the levels of hnRNP A2 bound to the transcript (Figure 5E, lanes 5 and 6; Figure 5F–G). CBF-A therefore interacts primarily with the RTS of the *Prm2* mRNA, although other sites may be contacted too.

We conclude that *in vitro*, p37 is the primary RTS binding factor and that p42 and hnRNP A2 can associate with the same RTS element. Interestingly, although being associated with the endogenous *Prm2* mRNA (Figure 4D), p42 and hnRNP A2 do not efficiently interact with the full-length *Prm2* mRNA synthesized by in vitro transcription, suggesting a fundamentally different mode of binding to the transcript in comparison to p37.

### p37 and p42 target different stages of the *Prm2* mRNA transcript

**Figure 6:**
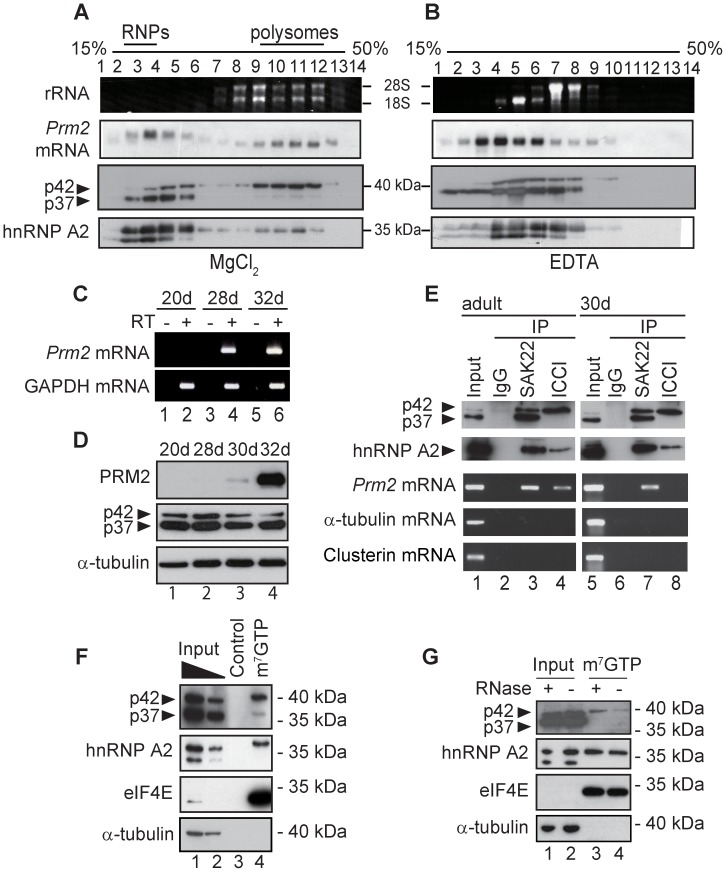
The translationally active *Prm2* mRNA associates with the p42 CBF-A isoform. (**A–B**) Fractionation of adult mouse testicular lysates on a 15–50% continuous sucrose gradient in the absence (A) or presence (B) of EDTA. Fractions were analyzed on Northern blots for the *Prm2* mRNA and on immunoblots for CBF-A (SAK22) and hnRNP A2. (**C–D**) The *Prm2* mRNA is translationally inhibited in postnatal day −28 and −30 mouse testis. (**C**) Developmental expression of *Prm2* mRNA in mouse testes. Total RNA was collected from mouse testis of 20, 28, 32 postnatal day of age (20–32 d), and analyzed by RT-PCR. RT, reverse transcriptase. (**D**) Developmental expression of PRM2 and CBF-A in mouse testes. Cytoplasmic testicular extracts from 20, 28, 30, 32 d mice were analyzed on immunoblots for PRM2, CBF-A (SAK22) and tubulin. (**E**) RIPs from adult and 30 d mouse testicular extracts. Cytoplasmic fractions were incubated with SAK22, ICCI or control anti-mouse IgGs. Immunoprecipitated fractions were analyzed on immunoblots for CBF-A (SAK22) and hnRNP A2, and by RT-PCR with primers amplifying the *Prm2*, α-tubulin or clusterin cDNAs. (**F–G**) The CBF-A p42 isoform associates with the 5′ mRNA cap complex. Cytoplasmic fraction from adult mouse testis was incubated with 7-methyl-GTP (m^7^GTP)-Sepharose or protein G-Sepharose (control). Bound proteins were analyzed on immunoblots for CBF-A (SAK22), hnRNP A2 and eIF4E and α-tubulin. (F) Lanes 1, 2 respectively 2% and 1% input. (G) Lane 1 and 2 are 2% input. Where indicated, testis lysates were pre-incubated with RNase A prior to incubation with the beads.

Since translation of the *Prm2* mRNA is temporally regulated, we next investigated whether CBF-A has a role in the *Prm2* mRNA translation, by monitoring the distribution of CBF-A in polysome profiles. Mouse testicular homogenates were fractionated in 15–50% continuous sucrose gradient, and each fraction was analyzed by Northern blotting for the *Prm2* mRNA and by immunoblotting for CBF-A (SAK22) and hnRNP A2. The distribution of rRNA shows that polysomes were sedimented at the higher density fractions (Figure 6A). As is the case for stored mRNAs [28], [39], the *Prm2* mRNA was detected in both free ribonucleoproteins (RNPs) and polysome fractions (Figure 6A). The molecular size of *Prm2* mRNA in polysome fractions was smaller than that in RNP fractions (Figure 6A), which is consistent with the fact that the translationally active *Prm2* mRNA is partially de-adenylated [28]. When the same fractions were analyzed on immunoblots for CBF-A, we found that both p37 and p42 co-sedimented with the RNP-containing fractions but only p42 was in the polysomes-rich fractions (Figure 6A). hnRNP A2 displayed a similar distribution to p42, co-sedimenting with both RNPs and polysome fractions (Figure 6A). A yet uncharacterized testis-specific hnRNP A2 variant was also found in the RNP fraction (Figure 6A). To determine whether the sedimentation of p42 and hnRNP A2 in the polysome fractions is due to their true association with polysomes, we added EDTA in the homogenate to dissociate ribosomal subunits (Figure 6B). EDTA eliminated rRNA and *Prm2* mRNA from the fractions eluting at the bottom of the gradient. Remarkably, we observed shifts of p42 and hnRNP A2 towards lighter sucrose fractions (Figure 6B). These results collectively show that p42 and hnRNP A2 associate with a translationally active form of the *Prm2* transcript in polysomes.

Juvenile mouse testes exhibit a very low proportion of elongating spermatids than adult mouse testes, and the majority of the *Prm2* mRNA in the early developmental stages is kept in a translationally repressed form [28]. Indeed, when we analyzed developmental expression of the *Prm2* mRNA and PRM2 in 20-, 28-, 30- and 32-dpp (days postpartum) mouse testes, we detected expression of the *Prm2* mRNA as from 28-dpp mouse testis, but significant levels of PRM2 expression were detected as from 32-dpp mouse testes (Figure 6C–D). We also confirmed that both CBF-A splice variants were present in the cytoplasmic fractions at all developmental stages (Figure 6D). Based on these observations, we next performed RIP analysis using 30 d mouse testes, to evaluate whether p42 associates with the translationally active *Prm2* mRNA. Testicular extracts from adult and 30-dpp mice were subjected to immunoprecipitations with the SAK22 and ICCI antibodies. Analysis on immunoblots confirmed that SAK22 co-precipitated p37, p42 as well as hnRNP A2 (Figure 6E, lanes 3 and 7), and the p42-specific ICCI antibody precipitated p42 and hnRNP A2 (Figure 6E, lanes 4 and 8) from both adult and 30-dpp testicular lysates. We next isolated total RNA from each of the immunoprecipitated fractions and reverse-transcribed with oligodT primers. The cDNA was analyzed by PCR with primers amplifying *Prm2*, α-tubulin and clusterin cDNAs. We found that the *Prm2* mRNA was co-immunoprecipitated with SAK22 from both adult and juvenile testicular cytoplasmic extracts (Figure 6E, lanes 3 and 7). In contrast, the p42-specific ICCI antibody co-precipitated the *Prm2* transcript from adult mouse testicular lysates but not from the extracts prepared from 30-dpp mouse testes under the same amplification conditions (Figure 6E, cf lanes 4 and 8). Since the *Prm2* mRNA was precipitated by SAK22, but not ICCI, this suggests that in juvenile mouse testis only p37 is associated with the *Prm2* mRNA while translationally inactive. Conversely, SAK22 precipitated the *Prm2* mRNA from adult testes, but since p37 is not found in the polysome fraction, this suggests that only p42 is associated with the *Prm2* mRNA engaged in translation.

### The p42 isoform interacts with the mRNA 5′ cap binding complex

We next examined whether p37, p42 and hnRNP A2 associate with the 5′ mRNA cap complex. For this purpose we incubated testicular cytoplasmic lysates with immobilized 7-methyl-GTP-cap analog beads (m^7^GTP beads). Bound proteins were resolved by SDS-PAGE and analyzed on immunoblots. We found that both p42 and hnRNP A2 were recovered among the cap-associated proteins whereas p37 was not enriched in the bound fraction (Figure 6F). As expected, the cap binding protein eIF4E was also among the cap-bound proteins (Figure 6F). Tubulin was not detected in the m^7^GTP beads bound fraction and none of the proteins analyzed was co-precipitated with the control beads (Figure 6F). To test whether the association of p42 is RNA-dependent, we treated testicular lysates with RNaseA prior to incubating with m^7^GTP beads. Analysis of bound fraction on immunoblots showed that co-precipitations of p42 and hnRNP A2 with m^7^GTP bead were not affected by the RNase treatment (Figure 6G). These results suggest that both p42 and hnRNP A2 associate with the translation machinery through direct protein-protein interactions.

Taken altogether, the above findings and previous results indicate that p37 targets the RTS of a translationally inactive form of the *Prm2* mRNA, whereas p42 binds to the RTS and 5′ cap binding complex of a translationally active form of the transcript.

### PRM2 expression levels are reduced in Hnrnpab−/− mouse testes

**Figure 7:**
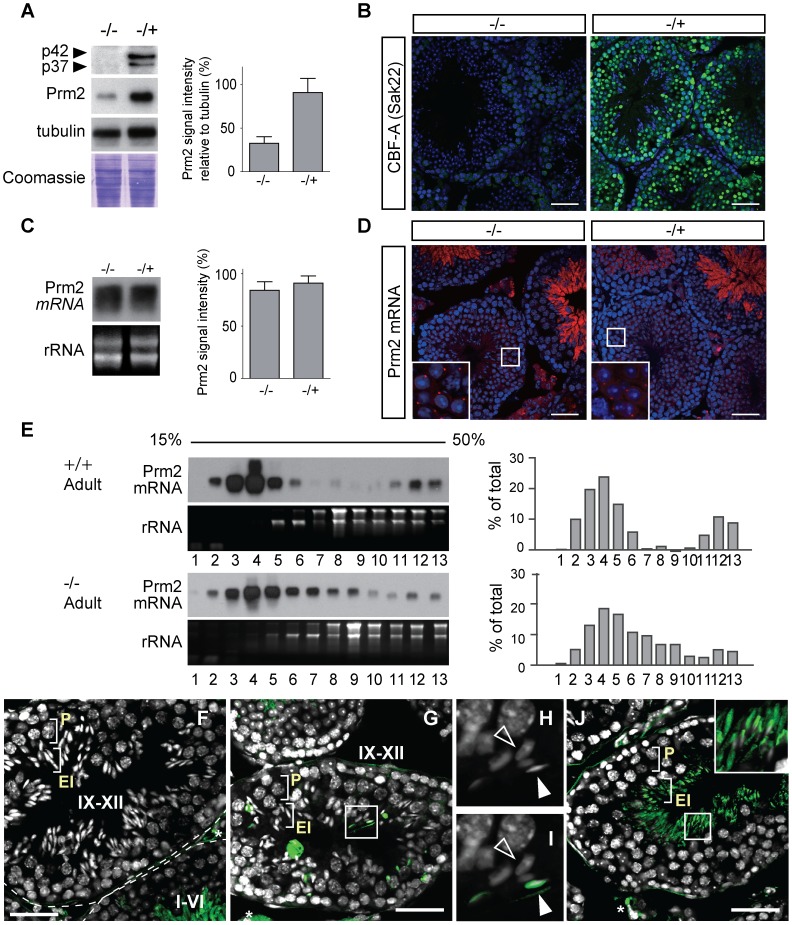
The *Prm2* mRNA expression is regulated by CBF-A at the translational level during spermatogenesis. (**A**) Immunoblots of CBF-A (Sak22), PRM2 and tubulin on Hnrnpab^+/−^ and Hnrnpab^−/−^ testis lysates. Total proteins were stained with Coomassie brilliant blue and shown as loading control. Densitometric analysis of the signals in the immunoblots was performed on 3 individual animals per genotypes. Data represent mean +/−SE ^*^ *p*<0.05 student t-test. (**B**) Immunostaining for CBF-A on Hnrnpab^+/−^ and Hnrnpab^−/−^ testis sections. Bar, 50 µm. (**C**) Northern blotting analysis for the *Prm2* mRNA on total RNA from Hnrnpab^+/−^ and Hnrnpab^−/−^ mouse testis. Densitometric analysis was on 3 individual animals of each genotype, and shown as mean +/− SE. (**D**) *In situ* hybridization on Hnrnpab^−/−^ and Hnrnpab^+/−^ testis sections showed no differences on *Prm2* mRNA expression. Bar, 50 µm. The inset shows a magnified image of the marked area. (**E**) Fractionation of testicular lysates from adult Hnrnpab^+/+^ and Hnrnpab^−/−^ mice on a 15–50% continuous sucrose gradient. Fractions were analyzed on Northern blots for the *Prm2* mRNA. (**F**) Testis section of Hnrnpab^+/−^ stained with a PRM2 antibody (Green) and DAPI (gray). PRM2 was not detected in the seminiferous tubules of stage IX–XII, in which pachytene spermatocytes (P) and elongating spermatids (EL) are contained. (**G**) Testis section from Hnrnpab^−/−^ stained with a PRM2 antibody (Green) and DAPI (gray). PRM2 signal was detected in some nuclei of elongating spermatids. (**H and I**) Magnified image of marked area in (G). DAPI staining shows that chromatin of PRM2-positive nuclei (white arrow head) appears more condensed than in PRM2-negative nuclei (blank arrow head). (**J**) Testis section of Hnrnpab^−/−^ stained with a PRM2 antibody (Green) and DAPI (gray). The seminiferous tubule was categorized as stage IX–XII, because it does not contain round-spermatid layer (see Figure S7), but nuclei of elongating spermatids appear prematurely condensed and positive for PRM2 signals. * denotes background staining of Leydig cells with the PRM2 antibody. Bars, 50 µm.

In order to examine whether CBF-A plays a role in the *Prm2* mRNA regulation in vivo, we analyzed the *Prm2* mRNA and PRM2 expression in the testis of the recently established CBF-A knockout mouse, referred to as Hnrnpab^−/−^ [31]. Using the SAK22 and ICCI antibodies, we confirmed that neither p37 nor p42 are present in the testes of homozygous mice (Figures 7A–B; Figure S5). Analysis of the PRM2 levels on immunoblots of testicular lysates from the Hnrnpab^−/−^ mouse testis revealed considerable decrease in the PRM2 expression in comparison to testicular lysates from heterozygous mice (Figure 7A). Quantification of the PRM2 levels relative to tubulin showed an average of 65% reduction in the expression of PRM2 in the Hnrnpab^−/−^ testis (n = 3 individuals per genotype, *p*<0.05 significant difference in the Student's t-test) (Figure 7A). We did not detect any significant differences in the progression of spermatogenesis in the seminiferous tubules or in the number of elongating and elongated spermatids, and epididymal sperm between Hnrnpab^+/−^ and Hnrnpab^−/−^ mice, suggesting that the reduction of PRM2 is not due to the arrest or cell death of spermatogenic cells (Figure S6). Next, we examined expression and localization of *Prm2* mRNA in testicular cells of Hnrnpab^−/−^ and Hnrnpab^+/−^ mice. Northern blotting analysis showed that the *Prm2* mRNA levels are not affected in the absence of CBF-A (Figure 7C). *In situ* hybridization of *Prm2* antisense probe revealed specific staining in the Hnrnpab^−/−^ testis, with similar intensities and localization patterns as in the Hnrnpab^+/−^ testis (Figure 7D). Similar to the wild-type mice (Figure 2), the *Prm2* mRNA was detected in chromatoid bodies of round spermatids (insets in Figure 7D), and in the cytoplasm of elongating and elongated spermatids of both Hnrnpab^−/−^ and Hnrnpab^+/−^ mouse testis (Figure 7D). These results raised the possibility that reduction of PRM2 in the CBF-A knockout mice is regulated at the translational level. This view is supported by polysome analysis performed on testicular lysates obtained from adult Hnrnpab^−/−^ mouse testis where we revealed a significant reduction in the *Prm2* mRNA levels in the translation-active fractions (Figure 7E). Thus, it is suggested that CBF-A contributes to the efficiency of the *Prm2* mRNA translation.

We also investigated if CBF-A has a role in translational repression of *Prm2* mRNA during spermatogenesis. Immunohistochemistry analysis performed on testicular sections from Hnrnpab^+/−^ and Hnrnpab^−/−^ mice with an anti-PRM2 antibody revealed that some spermatids express PRM2 at earlier stages of spermatogenesis. As shown in figure 7G–J, a subset of elongating spermatids in the seminiferous tubules of stage IX–XII are PRM2 positive. Further, the nuclei appeared more condensed than in PRM2-negative cells (Figure 7H–I). This peculiar expression pattern for PRM2 was observed in 38% of the stage IX–XII tubules in the Hnrnpab^−/−^ mice (11 out of 26 seminiferous tubules) whereas the PRM2 signal was visible only from elongated spermatids in the seminiferous tubules stage I–VI in Hnrnpab^+/−^ mice (Figure 7F), as previously shown in wild-type mice [23]. These results suggest that CBF-A is required to maintain the translationally repressed status of the *Prm2* mRNA in the cytoplasm of elongating spermatids during spermatogenesis. We conclude that repression of the *Prm2* mRNA translation is impaired in Hnrnpab^−/−^ mice.

Immunoblots of testes lysates from Hnrnpab^−/−^ mice revealed that the PRM1 and the Tnp2 protein levels are also down-regulated in the absence of CBF-A (Figure S7). On the other hand, Acrv1, Tnp1, ACT and tubulin levels were not affected (Figure S7). Consistent with these observations, RIP analysis with the CBF-A antibodies SAK22 and ICCI showed interactions of CBF-A with the Prm1 and Tnp2 transcripts but not with the Acrv1, Tnp1, ACT and tubulin transcripts which were not enriched in the immunoprecipitated fractions (Figure S7). These results suggest that CBF-A probably regulates testicular transcripts other than the *Prm2* mRNA, in a gene-specific manner.

**Figure 8:**
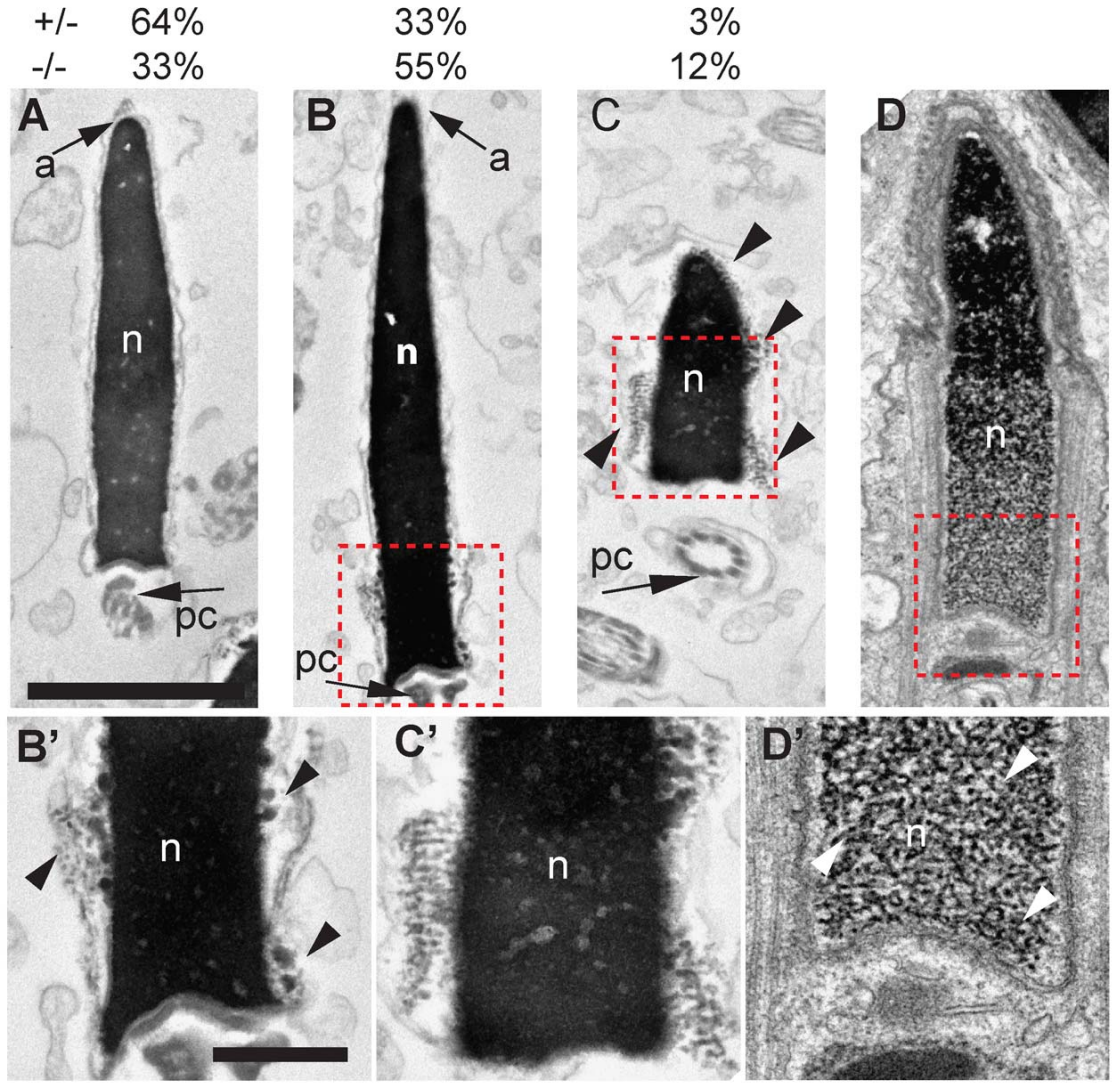
Transmission electron micrographs of sperm heads from the epididymis from Hnrnpab^−/−^ and Hnrnpab^+/−^ mice. (A) Normal sperm from Hnrnpab^−/−^ mouse showing the nucleus (n) revealed like an evenly stained black mass (due to high electron density) that contains the highly compacted DNA, surrounded by the nuclear envelope. The acrosome (a) located in the anterior half of the head and the proximal centriole (pc) in the neck of the sperm. (B) Abnormal sperm of Hnrnpab^−/−^ mice with fibers extending out of the main mass of local regions of nucleus (inset in B and B′). (C) Abnormal sperm of Hnrnpab^−/−^ mice with fibers extending out of the main mass around the nucleus (inset in C and C′). (D) Elongated spermatid at step 13 of spermatogenesis of wild-type mice. Posterior part of DNA, which is less packed than in anterior region, was observed as fibers (inbox in D and D′). Scale bar is 2 µm in A–D and 500 nm in B′–D′.

PRM2, PRM1 and TNP2 are essential proteins for DNA compaction in sperm nuclei [40]. To examine if the altered expression of these proteins in the Hnrnpab^−/−^ mouse affects spermatogenesis, we performed electron microscopic analysis of the epididymal sperm of CBF-A-deficient mice (Figure 8). We observed that 12% of sperm from Hnrnpab^−/−^ mice (14/117) display an abnormal structure with fibers that protrude out of the main black/dense DNA mass in the nucleus. This abnormality was detected in only 3% of sperm in the testis of Hnrnpab^+/−^ mice (3/102). The appearance of the fibers is similar to that of chromatin DNA observed in step10 spermatids, in which DNA compaction is under progress. This suggests that the abnormal sperms are defective in the compaction of DNA. We also detected a mild compaction defect in 55% (64/117) of Hnrnpab^−/−^ sperm (vs. 33%, 34/102 in Hnrnpab^+/−^ sperm). We conclude that translation regulation of the *Prm2* mRNA and possibly other testicular transcripts by CBF-A is required for DNA compaction in the sperm nucleus.

## Discussion

We report here that during spermatogenesis, the newly synthesized *Prm2* mRNA translocates and localizes to the chromatoid body and cytoplasm of round and elongating spermatids until it is targeted to polysomes for translation. To the best of our knowledge, our data provide first direct evidence that a translationally regulated transcript transits through the chromatoid body during spermatogenesis, underscoring the importance of spatial and temporal regulation of the *Prm2* mRNA throughout spermatogenesis. These results also emphasize that the translationally inactive *Prm2* mRNA is not only present in chromatoid body but it is also diffusely localized in the cytosol of round and elongating spermatids 41,42.

Several arguments indicate that the hnRNP CBF-A plays a role in the regulation of *Prm2* mRNA expression. We show in vivo evidence that CBF-A co-localizes with the *Prm2* mRNA in chromatoid bodies. In elongating spermatids, when chromatoid bodies are structurally and functionally transformed [43], CBF-A and the *Prm2* mRNA are both dispersed in the cytosol. This result is compatible with the distribution of CBF-A in polysome profiles as CBF-A co-sedimented with the *Prm2* mRNA in RNP and polysome fractions, enriched in chromatoid body and cytosol, respectively [44]. Furthermore, CBF-A was found to interact directly with *Prm2* mRNA via the RTS located in the 3′ UTR.

**Figure 9:**
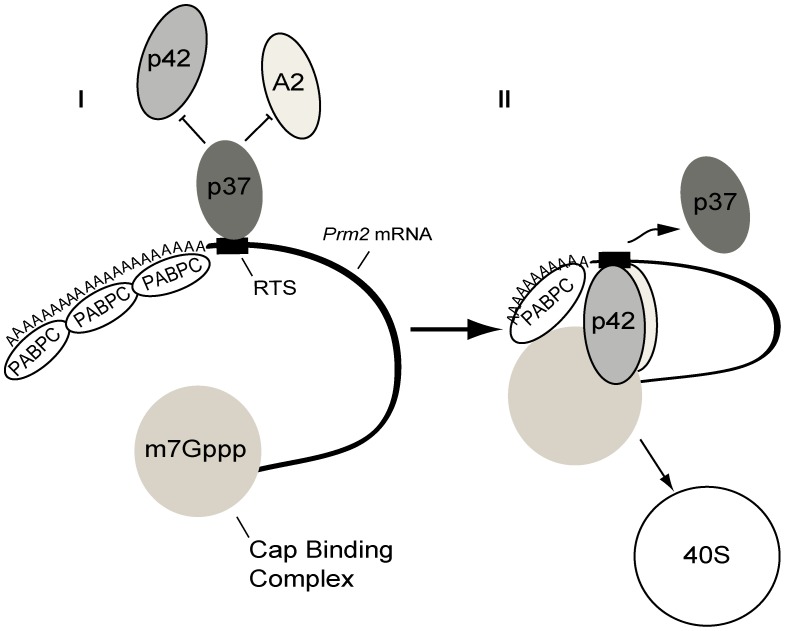
Speculative model for the role of CBF-A in translation regulation of the *Prm2* mRNA. We hypothesize that in early-step spermatids, p37 mainly binds to the RTS in the *Prm2* mRNA 3′UTR, leading to a translationally repressed configuration of the *Prm2* mRNA. In the cytoplasm of later step spermatids, p37 is released from the RTS through yet unknown mechanisms, and p42 is able to interact with the RTS and with the 5′ mRNA cap binding complex. This exchange may be regulated through cooperative association with hnRNP A2. We propose that the p37–p42 relay contributes to the temporal regulation of the *Prm2* mRNA translation during spermatogenesis.

We detected differences between the p37 and p42 CBF-A variants in RTS binding, polysome distribution, and in their abilities to associate with 5′ mRNA cap binding complex. p37 co-eluted with the translationally inactive *Prm2* mRNA and did not co-sediment with polysomes. In contrast to a p42-specific antibody, the pan CBF-A SAK22 antibody could co-immunoprecipitate the *Prm2* mRNA from d30 testes lysates where the majority of the *Prm2* mRNA is translationally repressed. Furthermore, although p37, p42 and hnRNP A2 are all present in the RNP fraction, RTS recognition by p37 competes away p42 and hnRNP A2 from interacting with the RTS element. Therefore, our hypothesis is that among the three RTS binding proteins, p37 can function during translational repression of the *Prm2* mRNA in round spermatids, contributing to keep the transcript in a translation inhibited state (Figure 9). In contrast to p37, we discovered that p42 co-fractionates with polysomes and p42 is not co-precipitated with the *Prm2* mRNA from d30 testis lysates where the *Prm2* mRNA is non-translating. Furthermore, p42 was co-precipitated with m^7^GTP beads in an RNA-independent manner. These observations indicate that p42 associates with the translationally active form of *Prm2* mRNA. Since in the absence of p37, p42 binds to the *Prm2* mRNA RTS, we propose that p42 contributes to establish a translationally active form of the transcript by primarily targeting 3′ UTR via the RTS element and 5′ mRNA cap binding complex (Figure 9). Selective association of p42 with the transcript may be facilitated by hnRNP A2 that does not compete with p42 for in vitro RTS binding (see Figure 5D) and enhances cap-dependent translation by interacting with the RTS [10]. Post-translational modifications on the CBF-A isoforms and hnRNP A2 may also contribute to modulate their recruitments to the transcript [45].

The analysis of testes from CBF-A knockout mice did not show alterations in the *Prm2* mRNA levels. The *Prm2* mRNA still associated with chromatoid bodies in testes lacking CBF-A, which suggests that CBF-A does not have a primary role in mRNA targeting to chromatoid bodies. However, we found significantly reduced PRM2 expression levels and early timing of *Prm2* mRNA translation. We hypothesize that p37 keeps the RNP in a translation-incompetent form and that the reduced levels of PRM2 in the CBF-A knockout mouse testis are due to the absence of the p42 splice variants.

p37 may stabilize the translation-incompetent *Prm2* mRNPs for intranuclear transport, and translocation to the chromatoid body and cytoplasm by interacting with motor proteins or factors that bridge the RNA with motor proteins such as the testis-brain RNA binding protein (TB-RBP), which is important for spermatogenesis and interacts with KIF17b [46], [47]. Following p37 release from the RTS, in our working model the 3′UTR undergoes remodeling and the RNP becomes transport-incompetent. This mechanism occurs concomitantly with shortening of the poly (A) tail and association of translation factors that promote formation of the circularized, translationally active *Prm2* mRNA [27]. The presence of p42 in polysomes and the direct interaction with both RTS and cap binding complex is consistent with this view. These results suggest that p42 recruitment to the transcript is required for the *Prm2* mRNA to engage the translation machinery. mRNA-protein complexes are subjected to dynamic changes in protein composition until a distinct mRNP emerges in the cytoplasm to engage the translation machinery [3], [48]. The different properties of the CBF-A isoforms with respect to the *Prm2* mRNP biogenesis are consistent with this scenario. We therefore speculate that p42 recruitment mediated by the RTS occurs in response to yet unknown developmental cues, which affect the *Prm2* mRNA maturation and stabilization, and lead to de-repression of the transcript for localized translation. Remodeling of the 3′UTR may be facilitated by specialized RNA helicases such as DDX25, an essential posttranscriptional regulator of spermatogenesis [49]. Whether during spermatogenesis CBF-A regulates the *Prm2* mRNA in tandem with microRNA-dependent mechanisms is an intriguing hypothesis [50], [51], but remains to be proven. The UTRs of several testicular transcripts have however proved critical for expression during spermatogenesis. Translation of the *Prm1* mRNA is kept repressed by a specific translational control element (TCE) found in the 3′UTR [52]. Although we have not been able to identify conserved RTSs in the UTRs of the *Prm1* and *Tnp2* mRNAs, their translations are down-regulated in the CBF-A knockout mouse. We therefore speculate that both transcripts are targeted by CBF-A through potential RTS-like sequences.

Overall, the lack of CBF-A represses translation of certain testicular transcripts and leads to abnormal sperms which are defective in DNA compaction. Even though there is a possibility of subinfertility, the Hnrnpab^−/−^ can produce pups, which would possibly be due to heterogeneity of the phenotype among spermatids. Analysis of testis sections for PRM2 revealed a premature translation pattern in a subset of spermatids in the seminiferous tubules of the CBF-A knockout mouse. Altered PRM1 and PRM2 distributions were also observed in a mosaic pattern in a Pbrp knockout mouse [53]. An emerging scenario therefore suggests that CBF-A and Prbp may work in tandem for the regulation of both PRM2 and PRM1 during spermatogenesis. Although other regulatory functions at the protein level cannot be excluded, including the possibility that CBF-A contributes to the general stability of a subset of factors involved in spermatogenesis, we favor the model that both CBF-A splice variants are part of a novel relay mechanism that regulates translation of several testicular transcripts and it is required during spermatogenesis.

## Materials and Methods

### Ethics statement

All experimental procedures on mice were performed according to Karolinska Institute and Stony Brook University animal core facility guidelines for the care and use of laboratory animals.

### Antibodies

The human Fab monoclonal antibody against MVH was purchased from BD Biosciences. The rabbit polyclonal antibody against MIWI is from Cell Signaling. The rabbit polyclonal anti-histone H3, and mouse monoclonal anti-fibrillarin, rabbit polyclonal anti-ACT antibodies were from Abcam whereas the goat polyclonal anti-PRM2, goat polyclonal anti-Tnp2, rabbit polyclonal anti-Tnp1, mouse monoclonal anti-hnRNP A2/B1, rabbit polyclonal anti-eIF4E, rabbit polyclonal anti-Tom20, and goat polyclonal ACRV1 antibodies were purchased from Santacruz Biotechnology. The mouse monoclonal anti-PRM1 and anti-PRM2 antibodies are from Briar Patch Biosciences. Both rabbit (ICCI) and guinea pig (SAK22) polyclonal abs against CBF-A were previously described by Raju et al. (2008, 2011) [11], [12]. Control non-specific mouse IgGs were from Abcam.

### In situ hybridization

Digoxigenin (DIG)-labeled RNA probes were synthesized by in vitro transcription using DIG RNA labeling mix (Roche). PCR products of *Prm2* amplified from mouse testis cDNA (forward primer, 5′- ATG GTT CGC TAC CGA ATG AG; reverse primer, 5′- GGC AGG TGG CTT TGC TC) were cloned into pGEM-T vector (Promega) and used as a template for the in vitro transcription. For preparation of cryosections, mouse testes were fixed with a solution of 4% paraformaldehyde (PFA) in 1× PBS for 5 h at 4°C, incubated with 15% sucrose in 1× PBS for 5 h and 30% sucrose in 1× PBS overnight, and subsequently embedded in the OCT compound (Sakura Finetek). Before the hybridization step, 10 µm thick sections were mounted on glass slides and post-fixed with 4% PFA in 1× PBS. The sections were subsequently treated with 1 µg/ml Protease K in 10 mM Tris-HCl (pH.7.5) at 37°C for 5 min and acetylated by incubating slide for 10 min with 0.25% Acetic Anhydride in 1.0 M Triethanol amine HCl (pH.8.0). Slides were rinsed in 0.85% NaCl for 5 min, and then incubated overnight at 60°C with *Prm2* anti-sense or sense RNA probes diluted in hybridization buffer containing 10 mM Tris-HCl, pH 7.0, 50% formamide, 0.2 ng/ml tRNA, 10% dextran sulfate, 1× Denhardt's solution, 600 mM NaCl, 0.25% SDS and 5 mM EDTA. The sections were serially washed with 50% formamide in 2× SSC, 2× SSC and 0.2× SSC at 65°C, and incubated with anti-DIG antibody conjugated with Dylight568 (Jackson laboratory) after blocking with 1.5% blocking reagent (Roche). The sections were counter-stained with DAPI.

### Immunohistochemistry

Squash testis samples for immunostaining in the chromatoid body (Figure 1J; Figure S2B) were prepared as described [34]. Immuno-FISH (Figure 3) was performed on the tubule squash samples prepared as described in [54]. 10 µm cryosections were prepared as described above. For antigen retrieval [33], cryosections and squash preparations were washed with 1× PBS, treated with microwave for 5 min in 10 mM sodium citrate buffer, pH 6.0, and permeabilized with a solution of 0.3% Triton-X in 1× PBS for 5 min. Slides were blocked with a solution containing 2% BSA, 0.05% Triton-X in 1× PBS and then incubated for 1 hr with the anti-CBF-A antibodies ICCI or SAK22 and anti-MVH or anti-hnRNP A2 antibodies. After washing with a solution of containing 0.05% Tween in 1× PBS, slides were incubated with species-specific fluorophore-conjugated secondary antibodies (FITC-Donkey anti rabbit, Cy3-Donkey anti rabbit, Cy5-Donkey anti human and Alexa568-Donkey anti mouse) for 1 hr at room temperature. For analysis, slides of untreated or microwave-treated testis sections as well as squash testis samples were visualized by light microscopy or laser scanning microscopy using an LSM510 confocal microscope (Zeiss).

### Cloning, expression and protein purification

The full-length open reading frame encoding CBF-A p42 was amplified from mouse hnRNP A/B cDNA plasmid (MR226335, ORIGENE) using primer pairs as follows: forward primer, 5′- GCGC (Bgl II) ATG TCG GAC GCG GCT GAG G - 3′ and reverse primer, 5′- GCGC (EcoRI) TCA GTA TGG CTT GTA GTT ATT CTG - 3′. The PCR products were cloned into pCRII (Invitrogen), sequenced, and subcloned between BamHI and EcoRI sites of pGEX-4T-3 (GE Healthcare) for expression as glutathione S-transferase (GST)-tagged CBF-A. For the CBF-A p37 isoform, full-length GST-tagged CBF-A (gift of Tomas Leanderson, Lund University) was expressed from a pGEX plasmid vector according to manufacturer's instruction protocols (GE Healthcare).

### Tissue extraction and fractionation

1 adult mouse testis was homogenized in 1 ml lysis buffer [1× PBS containing 0.2% NP-40, 40 U RnaseOut (Roche) and the cOmplete Protease Inhibitor (Roche)] by 20 strokes in a Dounce homogenizer at 4°C. For fractionation, the homogenates were centrifuged for 10 min at 1000 g, and the supernatant was collected as cytoplasmic extract. For nuclei fraction, the pellet was washed once with lysis buffer and resuspended. After sonication cytosolic and nuclear fractions were centrifuged at 15000 g for 15 min. The supernatants were used in immunoblotting or RNA immunoprecipitation assays.

### Immunoprecipitation and RNA immunoprecipitation/RT-PCR

Lysates were pre-cleared with Protein A-Sepharose 4B conjugate (Zymed, Invitrogen), and incubated overnight with anti-CBF-A antibodies, control anti-mouse IgGs or without antibodies (mock experiments). The antibodies were subsequently precipitated with Protein A-Sepharose 4B conjugate (Zymed, Invitrogen) for 1 h under continuous agitation. Where indicated, protein extracts were treated with 100 µg/ml RNase A for 15 min at room temperature before incubation with antibodies. Precipitated samples were resolved by SDS-PAGE and analyzed on immunoblots with antibodies against CBF-A or hnRNP A2. For analysis of the RNA species associated with CBF-A, the RNA was extracted from both input and immunoprecipitated fractions using the TRI reagent as described in the manufacturer's protocol (Sigma) and reverse-transcribed using Superscript II (Invitrogen) and oligo (dT) primers (Invitrogen). An equal volume of RNA incubated without Superscript II was used as a negative control (RT-). The samples were then analyzed by semi-quantitative PCR with primers specific to *Prm2* (see above for primers sequences), α-tubulin (forward primer, 5′- TTC GTA GAC CTG GAA CCC AC; reverse primer, 5′- TGG AAT TGT AGG GCT CAA CC) and clusterin (forward primer, 5′- CTG GAG CCA AGC CGC AGA CC; reverse primer, 5′- GCA CTC CTC CCA GAG GGC CA). Quantifications of PCR products were performed over 3 independent experiments using the ImageJ software.

### 7-methyl-GTP affinity chromatography

These assays were performed as in [44]. Briefly, adult mouse testes were homogenized in 1× PBS supplemented with 0.2% NP40 and a cocktail of protease inhibitors (cOmplete™, Roche). 40 µl of a 1∶1 suspension of either 7-methyl-GTP (m^7^GTP)-Sepharose 4B (Amersham Biosciences) or protein G-Sepharose 4B (Zymed) were blocked with 2% BSA for 1 h at 4°C. Beads were subsequently incubated with 400 µl of cytoplasmic testes lysates overnight at 4°C with rocking. Beads were washed 3× with a solution containing 1× PBS supplemented with 0.2% NP-40. Bound proteins were eluted by heat denaturation in SDS-containing Laemmli buffer. Eluted proteins were resolved by SDS-PAGE and analyzed on immunoblots for CBF-A, hnRNP A2, eIF4E and tubulin. Where indicated, testicular lysates were treated with 100 µg/ml RNaseA before incubation with the m^7^GTP-Sepharose 4B beads.

### RNA-protein interaction assays

For RNA affinity chromatography, biotinylated RNA oligonucleotides Prm2 wtRTS (5′- GCCCUGAGCUGCCAAGGAGCCGUACUGAG) as well as the previously described scrambled MBP RTS sequence (5′- GGGAGCGGAGAAACAAGCACCGAACCCGCAACUGG) [11] were purchased from Thermo Fisher Scientific. Full-length wt Prm2 mRNA and Δ3′ UTR Prm2 mRNA were synthesized by in vitro transcription using Biotin 11-UTP (Roche). 10 µl of streptavidin-Sepharose (GE Healthcare) were incubated with 100 pmol of oligonucleotide or 2 µg of transcribed RNAs in 100 µl of 1× PBS containing 0.1% NP40 for 30 min, washed once with 1× PBS containing 0.1% NP40, and then incubated with 200 µl of mouse testis lysates. Bound proteins were resolved by SDS-PAGE and analysed on immunoblots with antibodies to CBF-A (SAK22) and hnRNP A2. The Prm2 wtRTS conjugated to streptavidin beads was also used for *in vitro* pull-downs with recombinant p37, p42 and hnRNP A2. Briefly, 10 µl of the RNA-conjugated beads were incubated with purified recombinantly expressed p37, p42 and/or hnRNP A2 at final concentrations of 150 nM. Incubations were performed in 100 µl volume and allowed for 60 min at 4°C under continuous agitation. Bound proteins were eluted by heat denaturation in SDS loading buffer, resolved by SDS-PAGE and analyzed on immunoblots with antibodies to CBF-A (SAK22) or hnRNP A2.

### Analysis of polysomes

Sucrose gradient fractionation was carried out according to Unhavaithaya et al (2009) [38]. Briefly, one testis from wild-type, Hnrnpab^+/−^ or Hnrnpab^−/−^ mice was homogenized in 1 ml lysis buffer [100 mM NaCl, 3 mM MgCl_2_, 20 mM HEPES (pH 7.5), 0.1% Triton, 1 mM dithiothreitol (DTT), cOmplet Protease Inhibitor (Roche), 20 units/ml RNaseOu (Invitrogen)], by 20 strokes in a Dounce homogenizer. The lysates were treated with cycloheximide, a translational elongation inhibitor, at a final concentration of 200 µM to stabilize polysomes. The lysate was then centrifuged at 1300 g at 4°C for 2 min to pellet nuclei and cell debris. The supernatant was immediately layered onto the top of an 8 ml gradient of 15–50% (w/w) sucrose dissolved in the lysis buffer. For EDTA treatments, MgCl_2_ normally present in all buffers was replaced with 20 mM EDTA. The gradient was centrifuged for 3 h at 150000 g at 4°C, and collected in 14 fractions. RNA was extracted from 150 µl of each fraction, resolved by agarose gel electrophoresis, and analyzed by ethidium bromide or Northern blotting with probes hybridizing to the *Prm2* mRNA (see above). Alternatively, proteins in remaining fractions were concentrated to a volume of 100 µl by TCA precipitation, and 15 µl of each fraction was resolved by SDS-PAGE and immunoblotted for CBF-A and hnRNP A2.

### Analysis of RNA and proteins in Hnrnpab^−/−^ mouse testes

Mice were perfused with PBS containing heparin and 0.1 mM PMSF, and tissues were placed on ice before dissecting into fresh ice cold buffer. Protein lysates were made from one testis, and total RNA was extracted by Trizol from the other one. For Western blotting, 16 µg of protein samples were heat-denatured in 2× Laemmli buffer, separated on a 15% SDS-containing gel and transferred to a PVDF membrane. For Northern blotting, 2 µg of total RNA were separated on a 1.2% agarose gel. For each analysis, materials were individually collected from 3 animals per genotype.

### Transmission electron microscopy (TEM) of epididymal sperm

Sperm was collected from *caput epididymis*, dispersed into 1× PBS and kept at 4°C for analysis. Samples were then fixed in cacodylate buffer containing 2.5% glutaraldehyde and 4% paraformaldehyde for 2 h at room temperature, rinsed three times with the same buffer and post-fixed with 2% osmium tetraoxide for 1 h at room temperature. Spermatozoa were pelleted stepwise at 9000 rpm in an ultracentrifuge. Pre-embedding staining was performed with 1% uranyl acetate followed by sample dehydration through graded ethanol solutions embedded in epoxic-resin durcupan and polymerized for 48 h at 60°C. 80 nm ultrathin sections were collected on formvar/carbon-coated one-slot cupper grids (Agar Scientific), contrasted with uranyl acetate and lead citrate before examination in a transmission electron microscope at 100 kV.

## Supporting Information

Figure S1 (**A**) Sequence alignments of the CBF-A splice variants p37 and p42. The epitopes used to generate the antibodies SAK22 and ICCI are highlighted [11]. (**B**) Confocal picture of mouse testes cryosections co-immunostained with the anti-CBF-A antibodies SAK22 and ICCI. Nuclei were stained with DAPI and shown in blue. In the merged image all channels are shown. Scale bar, 50 µm.
(TIF)

Figure S2 *In vivo* distribution of hnRNP A2 in mouse testes. (**A**) Overview of mouse testes cryosections co-immunostained with a monoclonal antibody to hnRNP A2/B1 (red) and with the rabbit polyclonal anti-CBF-A antibody ICCI (green). Following Immunostaining, sections were analyzed by confocal microscopy. Nuclei were stained with DAPI and shown in blue. In the merged image all channels are shown. Scale bar, 50 µm. (**B**) Co-immunostaining on squash preparations of testicular cells with a monoclonal antibody to hnRNP A2/B1 (red) and with the rabbit polyclonal anti-CBF-A antibody ICCI (green). Signals of CBF-A and hnRNP A2/B1 were found to co-localize in chromatoid bodies of round spermatids (see arrowhead). Scale bar, 10 µm.
(TIF)

Figure S3 Fluorescence *in situ* hybridization performed on wild-type mouse testis sections using the *Prm2* mRNA sense and antisense probes, revealed by confocal microscopy. The sense probe did not give any signal, supporting the specificity of the *Prm2* mRNA localization studies.
(TIF)

Figure S4 Overview of immuno-FISH staining on squash preparations of stage-selected seminiferous tubules. The stages of the seminiferous tubules were determined by the light absorption patterns as described by Kotaja *et al.* (2004) [54], and the seminiferous tubules around stages I–VI, VII–VIII and IX–XII were subjected to squash preparation and immuno-FISH staining. Cells were triple-stained with antisense probe for the *Prm2* mRNA (red), anti-PRM2 antibody (green) and anti-MVH antibody (white) as well as DAPI. Developmental steps of spermatids were indicated by white arrows. RS, round spermatids; ES, elongating or elongated spermatids. Scale bars, 15 µm.
(TIF)

Figure S5 Immunostaining of CBF-A (ICCI) and hnRNP A2/B1 on Hnrnpab^−/−^ and Hnrnpab^+/−^ testis sections. Sections were counter-stained with DAPI. Note that the CBF-A antibody ICCI gave no significant staining on Hnrnpab^−/−^ spermatogenic cells, whereas the hnRNP A2/B1 gave similar staining patterns on both Hnrnpab^−/−^ and Hnrnpab^+/−^ testis sections.
(TIF)

Figure S6 Overview of testis sections of Hnrnpab^−/−^ (**A**) and Hnrnpab^+/−^ (**B**). Sections were stained with a Stra8 antibody (red), which marks Preleptotene spermatocytes in Stage VII–VIII [57], H1t antibody (green), which stains mid-pachytene spermatocyte to early elongating spermatids [58], and DAPI (blue). (**C–E**) High magnification view of the seminiferous tubules of stage I–VI (C), VII–VIII (D), and IX–XII (F) in Hnrnpab^−/−^ testis. (**F–G**) Seminiferous tubules were categorized into three groups as stage I–VI, VII–VIII, and IX–XII, based on the presence or absence of round spermatids and Stra8 signal in the tubules. Percentage of each groups in the section of Hnrnpab^−/−^ and Hnrnpab^+/−^ were shown in panel G. Even though 3% of the Hnrnpab^−/−^ tubules were abnormal, with smaller cell number in the tubules or irregular combination of spermatogenic cells (as shown in Figure 7), most of the tubules were apparently normal and there were no significant differences in the percentage of each seminiferous tubule cycles. (**H**) Number of elongated spermatids per seminiferous tubule (stage IX–XII). (**I**) Number of elongated spermatids in the seminiferous tubules (stage I–VI), obtained in 5 different tubules in 10 µm cryosections. (**J**) Number of epididymal sperm of Hnrnpab^−/−^ and Hnrnpab^+/−^ mice. 1 drop of sperm cells was collected from *cauda epidydimis* into 0.3 ml 1× PBS, dispersed, and cell number was counted with a Hemocytometer.
(TIF)

Figure S7 Analysis of CBF-A target genes in mouse testis. (**A**) Immunoblots of Hnrnpab^+/−^ and Hnrnpab^−/−^ testis lysates. Numbers indicate the average of signal intensities of 3 different samples, shown as ratio against +/−. Tnp2, Transition protein 2. Acrv1, acrosomal vesicle protein 1. ACT, activator of CREM. (**B**) RIP analyses of testicular mRNAs on wild-type adult mice testis. Lane1, input; lanes 2–4, immunoprecipitated fractions by non-specific IgGs, SAK22, and ICCI, respectively.
(TIF)
