# Supplementary figures and images for: The Transacting Factor CBF-A/Hnrnpab Binds to the A2RE/RTS Element of Protamine 2 mRNA and Contributes to Its Translational Regulation during Mouse Spermatogenesis
Source: PLoS Genet. 2013 Oct 17;9(10):e1003858. doi: 10.1371/journal.pgen.1003858 (PMC3798277; doi:10.1371/journal.pgen.1003858)

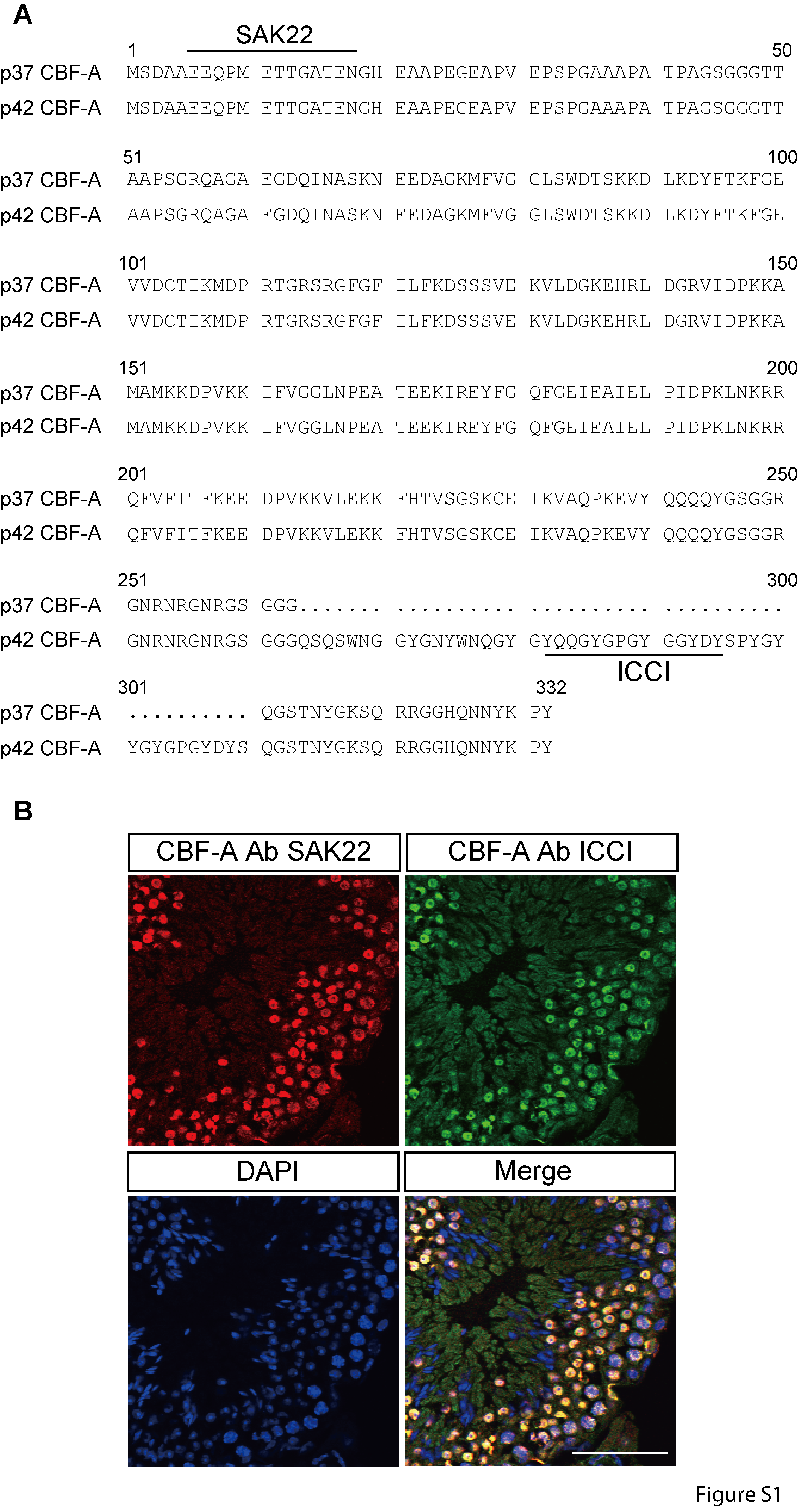

Supplement: Figure S1 — (A) Sequence alignments of the CBF-A splice variants p37 and p42. The epitopes used to generate the antibodies SAK22 and ICCI are highlighted [11]. (B) Confocal picture of mouse testes cryosections co-immunostained with the anti-CBF-A antibodies SAK22 and ICCI. Nuclei were stained with DAPI and shown in blue. In the merged image all channels are shown. Scale bar, 50 µm. (TIF) [file pgen.1003858.s001.tif]

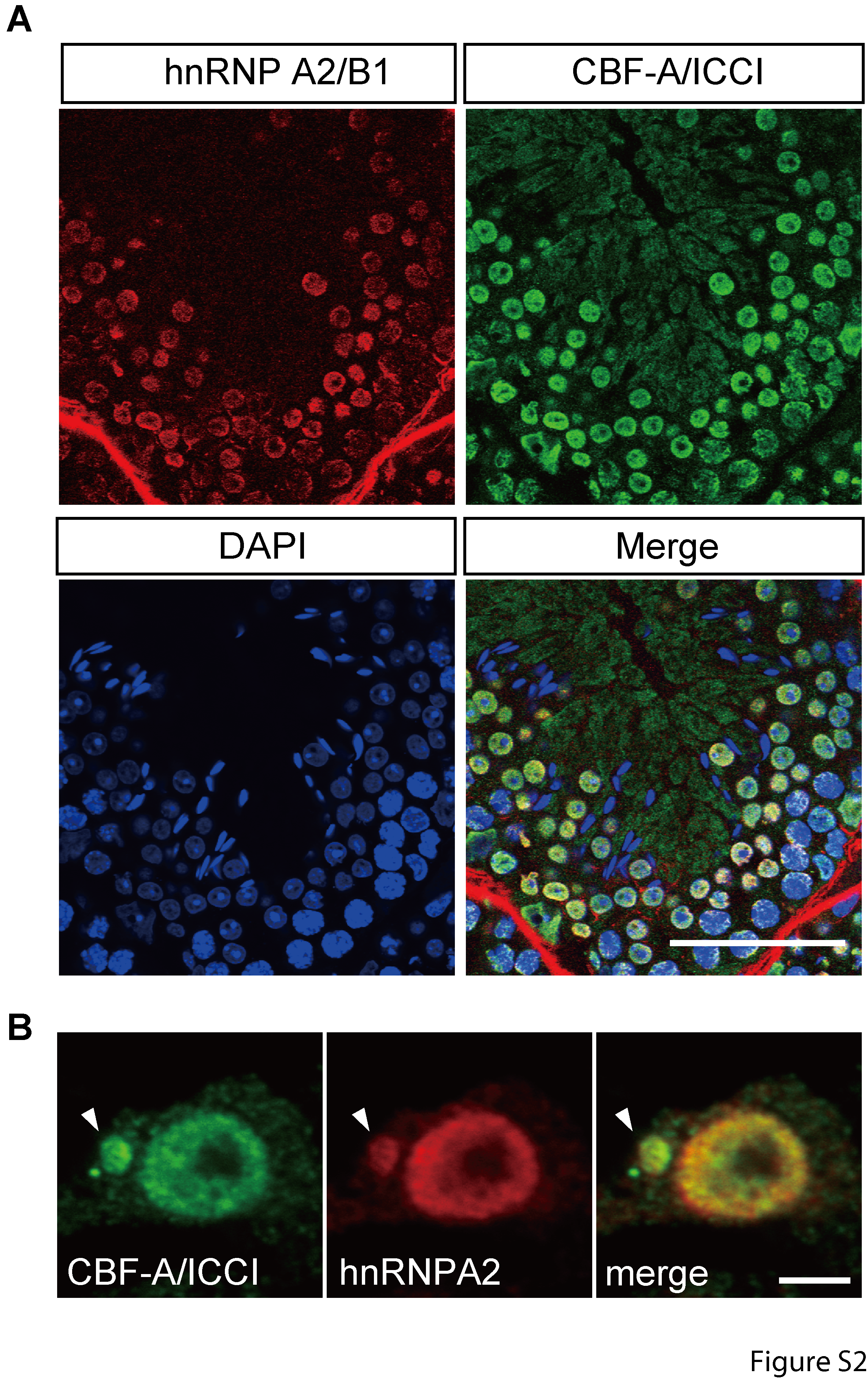

Supplement: Figure S2 — In vivo distribution of hnRNP A2 in mouse testes. (A) Overview of mouse testes cryosections co-immunostained with a monoclonal antibody to hnRNP A2/B1 (red) and with the rabbit polyclonal anti-CBF-A antibody ICCI (green). Following Immunostaining, sections were analyzed by confocal microscopy. Nuclei were stained with DAPI and shown in blue. In the merged image all channels are shown. Scale bar, 50 µm. (B) Co-immunostaining on squash preparations of testicular cells with a monoclonal antibody to hnRNP A2/B1 (red) and with the rabbit polyclonal anti-CBF-A antibody ICCI (green). Signals of CBF-A and hnRNP A2/B1 were found to co-localize in chromatoid bodies of round spermatids (see arrowhead). Scale bar, 10 µm. (TIF) [file pgen.1003858.s002.tif]

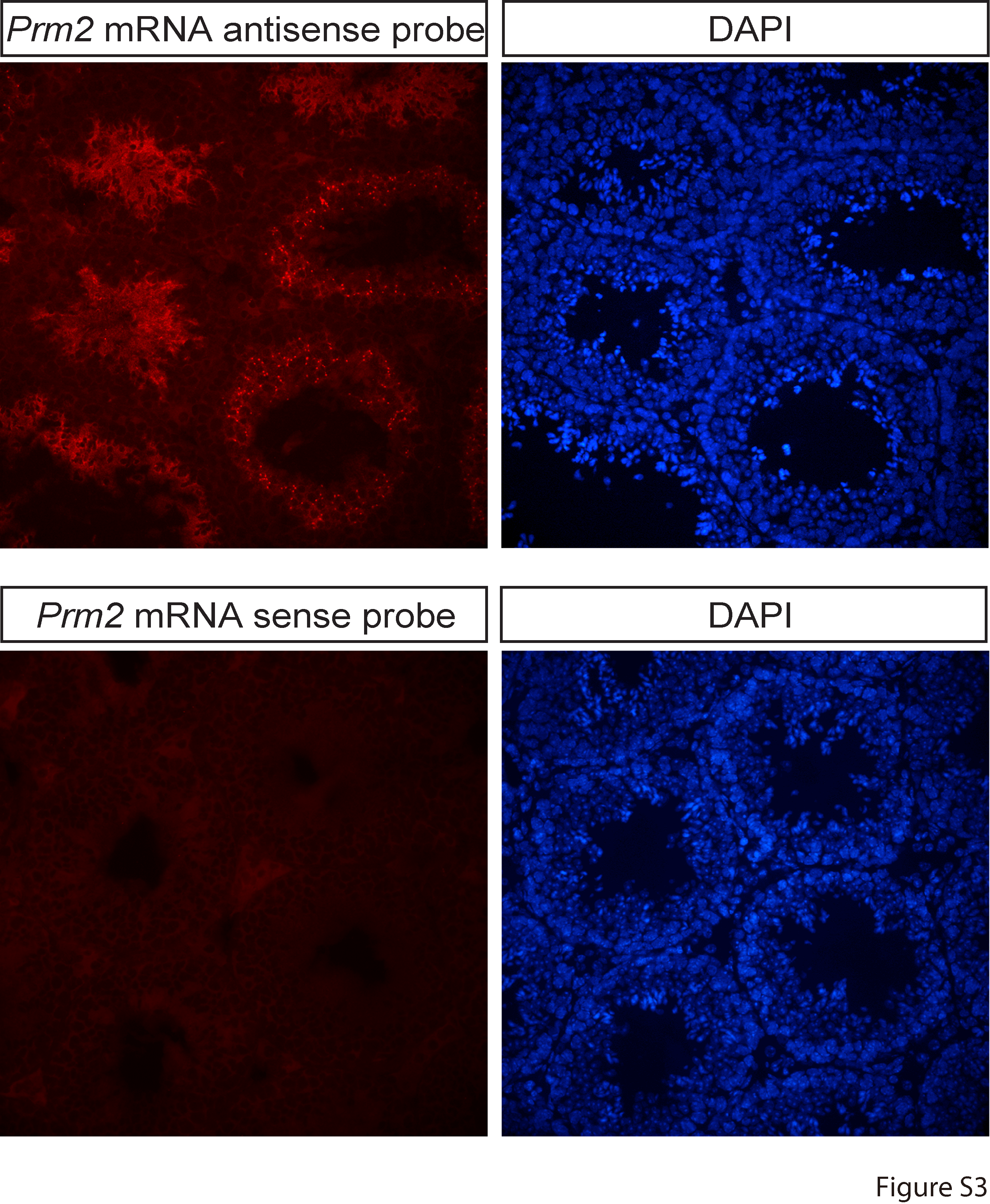

Supplement: Figure S3 — Fluorescence in situ hybridization performed on wild-type mouse testis sections using the Prm2 mRNA sense and antisense probes, revealed by confocal microscopy. The sense probe did not give any signal, supporting the specificity of the Prm2 mRNA localization studies. (TIF) [file pgen.1003858.s003.tif]

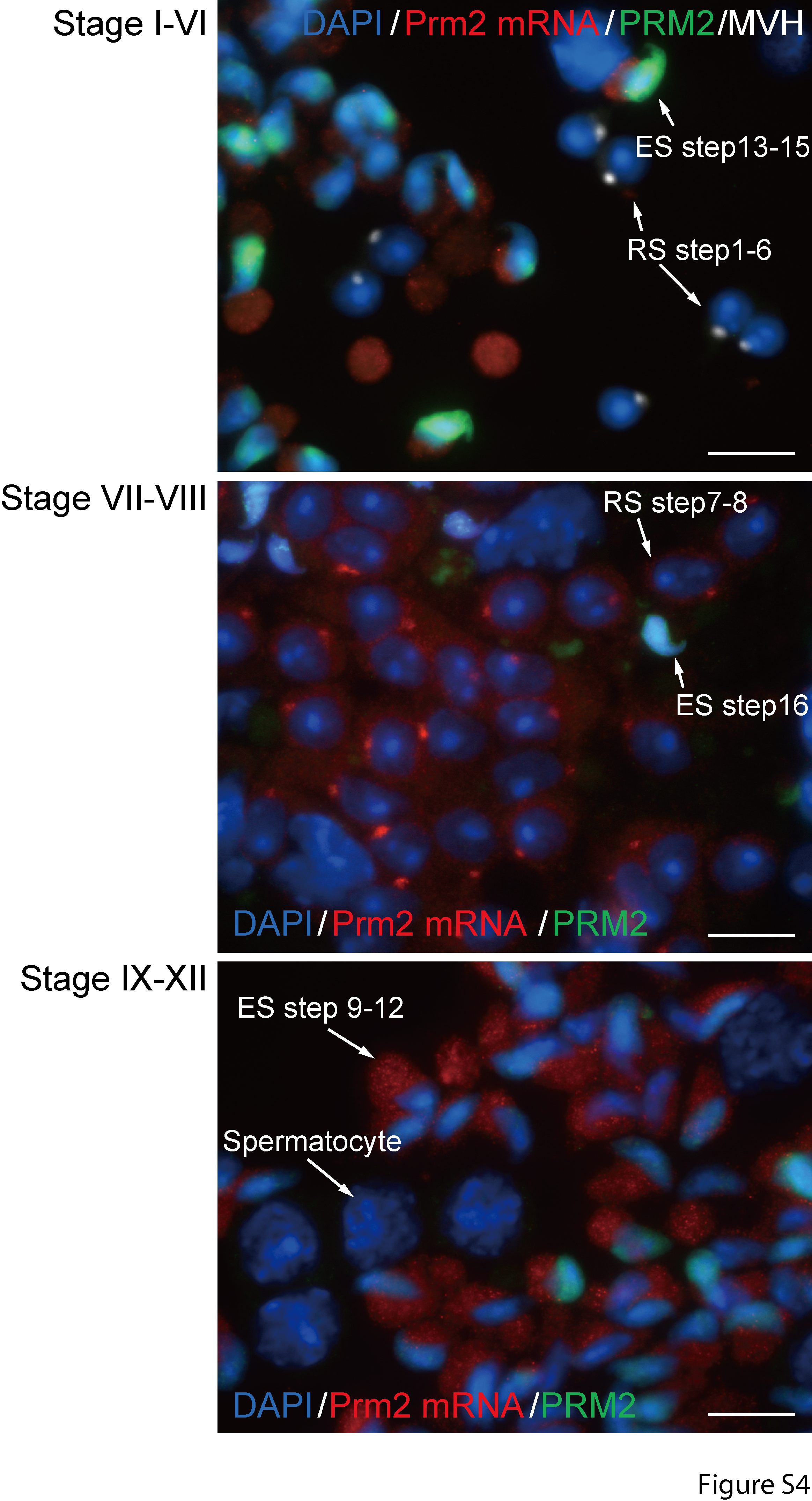

Supplement: Figure S4 — Overview of immuno-FISH staining on squash preparations of stage-selected seminiferous tubules. The stages of the seminiferous tubules were determined by the light absorption patterns as described by Kotaja et al. (2004) [54], and the seminiferous tubules around stages I–VI, VII–VIII and IX–XII were subjected to squash preparation and immuno-FISH staining. Cells were triple-stained with antisense probe for the Prm2 mRNA (red), anti-PRM2 antibody (green) and anti-MVH antibody (white) as well as DAPI. Developmental steps of spermatids were indicated by white arrows. RS, round spermatids; ES, elongating or elongated spermatids. Scale bars, 15 µm. (TIF) [file pgen.1003858.s004.tif]

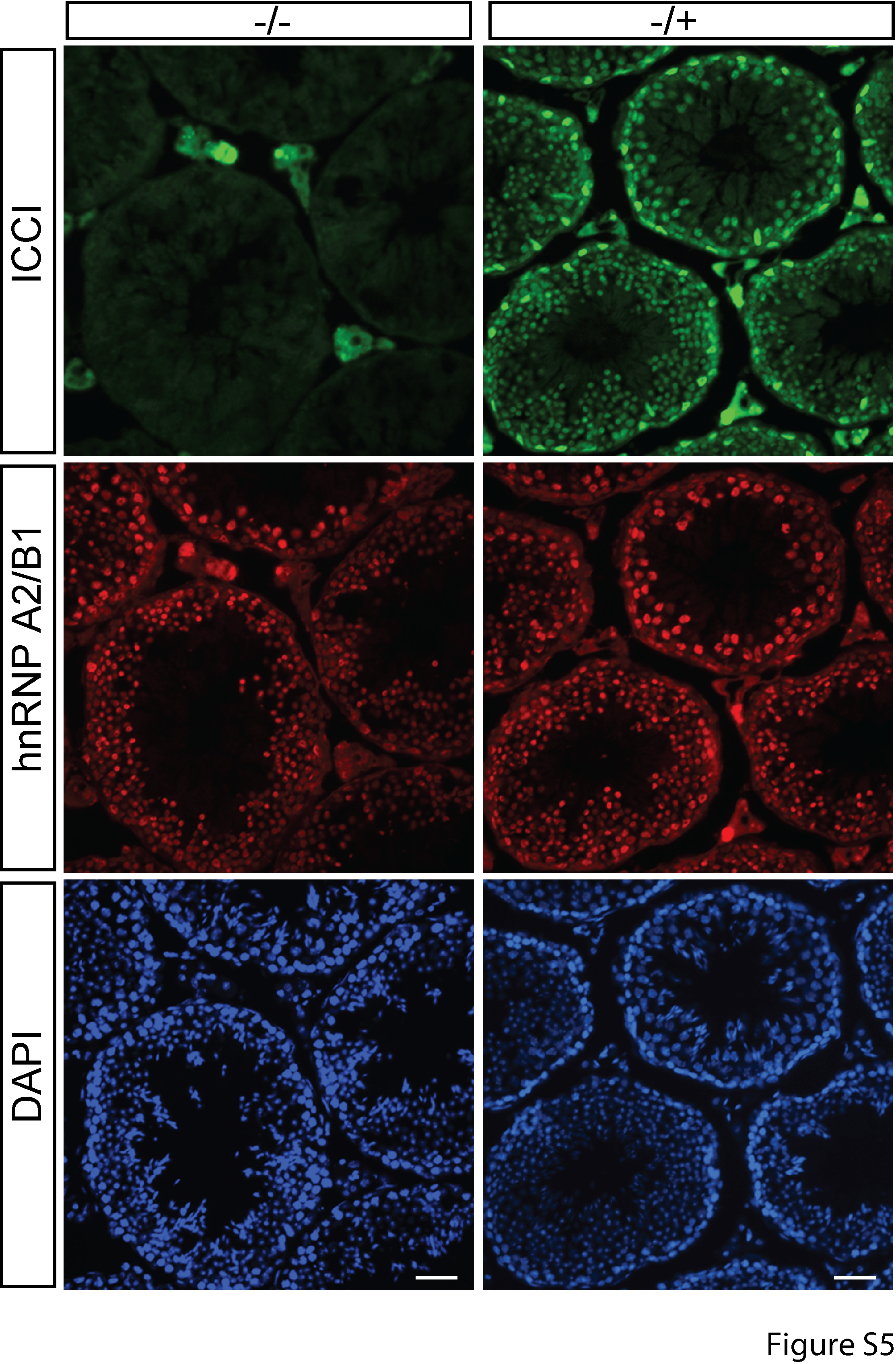

Supplement: Figure S5 — Immunostaining of CBF-A (ICCI) and hnRNP A2/B1 on Hnrnpab−/− and Hnrnpab+/− testis sections. Sections were counter-stained with DAPI. Note that the CBF-A antibody ICCI gave no significant staining on Hnrnpab−/− spermatogenic cells, whereas the hnRNP A2/B1 gave similar staining patterns on both Hnrnpab−/− and Hnrnpab+/− testis sections. (TIF) [file pgen.1003858.s005.tif]

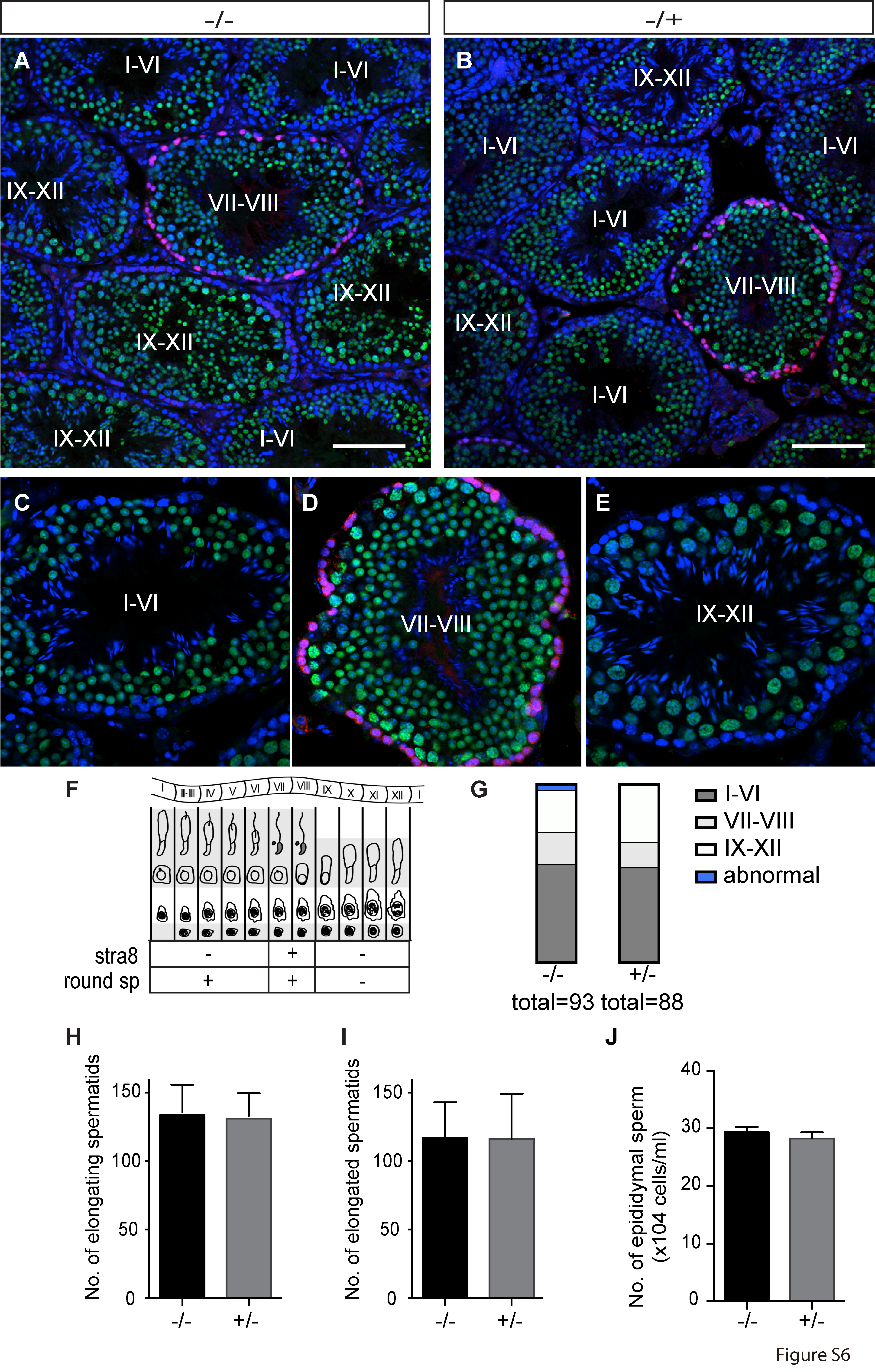

Supplement: Figure S6 — Overview of testis sections of Hnrnpab−/− (A) and Hnrnpab+/− (B). Sections were stained with a Stra8 antibody (red), which marks Preleptotene spermatocytes in Stage VII–VIII [57], H1t antibody (green), which stains mid-pachytene spermatocyte to early elongating spermatids [58], and DAPI (blue). (C–E) High magnification view of the seminiferous tubules of stage I–VI (C), VII–VIII (D), and IX–XII (F) in Hnrnpab−/− testis. (F–G) Seminiferous tubules were categorized into three groups as stage I–VI, VII–VIII, and IX–XII, based on the presence or absence of round spermatids and Stra8 signal in the tubules. Percentage of each groups in the section of Hnrnpab−/− and Hnrnpab+/− were shown in panel G. Even though 3% of the Hnrnpab−/− tubules were abnormal, with smaller cell number in the tubules or irregular combination of spermatogenic cells (as shown in Figure 7), most of the tubules were apparently normal and there were no significant differences in the percentage of each seminiferous tubule cycles. (H) Number of elongated spermatids per seminiferous tubule (stage IX–XII). (I) Number of elongated spermatids in the seminiferous tubules (stage I–VI), obtained in 5 different tubules in 10 µm cryosections. (J) Number of epididymal sperm of Hnrnpab−/− and Hnrnpab+/− mice. 1 drop of sperm cells was collected from cauda epidydimis into 0.3 ml 1× PBS, dispersed, and cell number was counted with a Hemocytometer. (TIF) [file pgen.1003858.s006.tif]

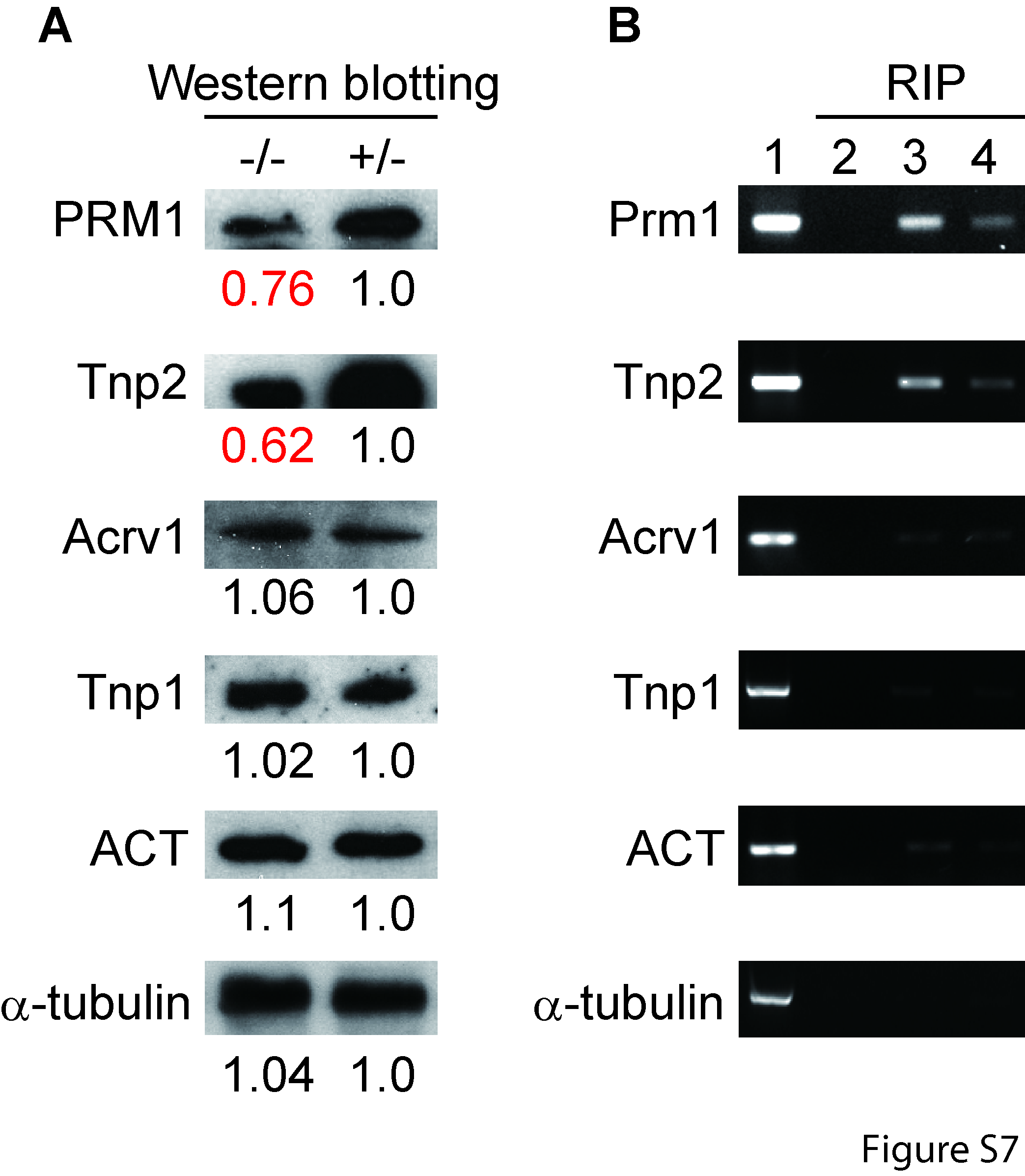

Supplement: Figure S7 — Analysis of CBF-A target genes in mouse testis. (A) Immunoblots of Hnrnpab+/− and Hnrnpab−/− testis lysates. Numbers indicate the average of signal intensities of 3 different samples, shown as ratio against +/−. Tnp2, Transition protein 2. Acrv1, acrosomal vesicle protein 1. ACT, activator of CREM. (B) RIP analyses of testicular mRNAs on wild-type adult mice testis. Lane1, input; lanes 2–4, immunoprecipitated fractions by non-specific IgGs, SAK22, and ICCI, respectively. (TIF) [file pgen.1003858.s007.tif]
